# Revision of the orchid bee subgenus *Euglossella* (Hymenoptera, Apidae), Part I, The decorata species group

**DOI:** 10.3897/zookeys.140.1923

**Published:** 2011-10-26

**Authors:** Ismael A. Hinojosa-Díaz, Michael S. Engel

**Affiliations:** Division of Entomology, Natural History Museum, and Department of Ecology & Evolutionary Biology, University of Kansas, 1501 Crestline Drive – Suite 140, University of Kansas, Lawrence, Kansas 66045, USA

**Keywords:** Hymenoptera, Apoidea, Anthophila, Euglossini, *Euglossa*, new species, taxonomy, orchid bees

## Abstract

*Euglossella*, one of the most distinctive subgenera of orchid bees of the genus *Euglossa*, is composed of two characteristic assemblages of species, one of them comprising bees bearing the strongly metallic integument trademark of the genus (*viridis* species group), and the other consisting of bees with a brown integument shaded with metallic iridescence (*decorata* species group). Here we provide the first of two parts of a revision of *Euglossella*, providing diagnostic definitions for the subgenus, the *decorata* species group, and all the species included therein. Six species are included in the *decorata* group, one new: *Euglossa* (*Euglossella) aurantia*, **sp. n.**; *Euglossa (Euglossella) apiformis* Schrottky, resurrected status; *Euglossa (Euglossella) decorata* Smith, revised status; *Euglossa (Euglossella) singularis* Mocsáry, revised status; *Euglossa (Euglossella) cosmodora* Hinojosa-Díaz and Engel; and *Euglossa (Euglossella) perpulchra* Moure and Schlindwein. *Euglossa meliponoides* Ducke and *Euglossa urarina* Hinojosa-Díaz and Engel are newly synonymized under *Euglossa decorata*, *Euglossa decorata ruficauda* Cockerell is synonymized under *Euglossa singularis*, and a neotype is designated for *Euglossa apiformis*.

## Introduction

Among orchid bees of the genus *Euglossa*, one of the most distinctive groups are those species of the subgenus*Euglossella*, with their tridentate mandibles, lamellate pronotal dorsolateral angles, slender mesobasitarsi, truncate ventral margins of the metabasitarsi, and scalene triangular metatibiae. This subgeneric assemblage was originally established by Perty (1833) under the generic name *Cnemidium*, a homonym, but renamed and more truly characterized by Moure (1967) to encompass those *Euglossa* in which the males have tridentate mandibles. Dressler (1978b) reinterpreted the subgenus by considering additional characters, most of them secondary sexual features of the males, making it a more coherent taxonomic unit. Hinojosa-Díaz (2008), when discussing the male genitalic morphology across *Euglossa*, gave an account of features that further contributed to the cohesiveness of *Euglossella* as a subgenus. Recent phylogenetic analyses based both on external morphology (Hinojosa-Díaz 2010, in prep.) and molecular data (Ramírez et al. 2010), situate *Euglossella* as a monophylelic entity sister to all other *Euglossa* *sensu lato*, either alone (morphology) or in a clade together with the subgenus *Dasystilbe* (molecular). Within *Euglossella* a clear distinction can be traced to group the species in two easily recognizable species groups. The first includes all those species that, as is the rule for all other *Euglossa* outside of *Euglossella*, have strongly and brightly metallic body integument, which is those species resembling *Euglossa (Euglossella) viridis* (Perty), type species of the subgenus. The second species group includes species characterized by a distinctive yellow-brownish coloration with secondary iridescence on the head and mesosoma, and an almost complete absence of metallic color on the metasoma, and includes thosetaxa resembling *Euglossa (Euglossella) decorata* Smith. Besides the morphological distinction, the *viridis* species group has a wide Neotropical distribution, from southern Mexico to southern Brazil, while the *decorata* species group is restricted to South America East of the Andes, in areas surrounding the Amazon Basin. A taxonomic revision of the *decorata* species group is here presented as the first of two parts dedicated to the subgenus *Euglossella*. Diagnoses for each recognized taxon are provided, along with detailed descriptions for four species – one of them proposed as new and another resurrected from synonymy – and two others with clarified status.

## Material and methods

Material examined in this study is deposited in the following collections: Division of Entomology, University of Kansas Natural History Museum, Lawrence, Kansas, USA (SEMC); Florida Museum of Natural History, University of Florida, Gainesville, Florida, USA(FLMNH); The Natural History Museum, London, United Kingdom (NHML); American Museum of Natural History, New York, New York, USA (AMNH); Museu Paraense Emílio Goeldi, Belém, Pará, Brazil (MPEG); Museu de Historia Natural, Universidade Federal de Minas Gerais, Belo Horizonte, Minas Gerais, Brazil (BHMH); Hungarian Natural History Museum, Budapest, Hungary (HNHM); Departamento de Zoologia, Universidade Federal do Paraná, Curitiba, Paraná, Brazil (DZUP); Zoologische Staatsammlung München, Munich, Germany (ZSSM); National Museum of Natural History (Smithsonian Institution), Washington, D.C., USA (USNM); Claus Rasmussen personal collection, Denmark (CRAS). The enumeration of specimens examined follows a detailed description of the label data, the information for each specimen enclosed by quotation marks (“”), each label separated by double slash symbols (//), and every row on individual labels separated by a semicolon in italics (;).

Morphological terminology in general follows that of Engel (2001), Michener (2007), and Hinojosa-Díaz (2008), while someprocedures for establishing metrics follow those of Brooks (1988). The species descriptions follow the overall format for other *Euglossa* species as presented by Hinojosa-Díaz and Engel (2007) and Hinojosa-Díaz et al. (2011).Photomicrographs were prepared using a Cannon EOS 7D digital camera and an Infinity K-2 long-distance microscope lens. Multilayer images were produced by using the software CombineZP.

## Systematics

### Genus *Euglossa* Euglossa

#### Subgenus Euglossella

Moure

http://species-id.net/wiki/Euglossella

Cnemidium Perty, 1833: 148, *nomen praeoccupatum* (*nec*Goldfuss, 1826). Type species: *Cnemidium viride* Perty, 1833, monobasic.
Euglossa (*Euglossella*) Moure, 1967: 401, *nomen novum pro Cnemidium* Perty, 1833. Type species: *Cnemidium viride* Perty, 1833, autobasic.

##### Diagnosis.

 Mid-sized metallic bees, with rather robust habitus; both sexes with tridentate mandibles and pronotal dorsolateral angles projected as acute prong or lamella (Fig. 3); female metabasitarsus trapezoidal with noticeably narrow distal margin (Figs 26, 46, 56, 65, 74); male mesotibia with two tufts, anterior tuft ellipsoidal, occupying about one-third of the outer tibial surface, posterior tuft rounded in a variety of shapes (Figs 24, 44, 54); male mesobasitarsus characteristically elongate and slender (Fig. 4), distal mesotarsomeres (specially second) unmodified; inner surface ofmale metafemur with ventral margin distinctively straight; male metatibia scalene triangular, metatibial organ slit basal and distal sections separated by a constriction distinctively narrower than width of contiguous basal section, basal section ellipsoidal, distal section separated from ventral margin of tibia by less than its own length (Fig. 6); ventral margin of inner metatibial surface with a blunt projection adjacent to spur attachment; male metabasitarsus roughly rectangular, ventral margin roughly straight in respect to sagittal body plane, appearing truncate and without noticeable projections of posterior margin. Eighth metasomal sternum of male with lateral edges of posterior section deeply invaginated, lobes strongly projected (Fig. 26); posterior margin of apical process of gonocoxite oblique (inner-posterior corner displaced posteriad) (Fig. 30); lateral area of gonostylar process of gonocoxite truncate; spatha surface with longitudinal striae (Fig. 30); dorsal sector of lateral section of gonostylus convex, covered with distinctive plumose setae, gonostylar ventral lobe thumb-like (Figs 33–34).

##### Key to species groups of *Euglossella*

**Table:** 

1	Integument of entire body strongly and brightly metallic blue, green, purple or reddish (e.g., figure 2); tegula metallic (usually same color as mesoscutum), never completely translucent (sometimes translucent on margins);metasomal terga usually with dense strong punctation	*viridis* species group
–	Integument of head and mesosoma with a dominant basal brown to dark brown color, shaded by a varying degree of metallic iridescence, particularly green, cyan, and coppery; integument of metasoma varying from golden-orange to dark brown with very faint metallic hue or iridescence (Fig. 1); tegula hyaline translucent with faint metallic hue; punctures on metasomal terga usually shallow.	*decorata* species group

### The *decorata* species group

**Recognition.** The bees of the *decorata* species group are easily recognizable from other *Euglossella* species mainly based on their integumental coloration. Species of the *decorata* group,unlike all other *Euglossa sensu lato*, have brown as the base color of their head and mesosoma, tinged with iridescence to different degrees but on close observation the underlying brown coloration can be seen. This integumental color feature can be appreciated more easily as it is expressedon the tegula, which in these bees is characteristically hyaline with no metallic coloration on it beyond some faint hue. The legs and the metasoma are practically devoid of metallic coloration, and can be of any color between yellow and very dark brown, although as for the tegula, they can have some faint hue. This rather distinctive coloration makes the species of the *decorata* species group appear at first sight similar to species of the genus *Melipona* (Apinae, Meliponini). The integumental sculpturing,especially on the metasoma, is rather shallow, contrasting with the usually strong punctures present on the metasomal terga of all other *Euglossella*. Additionally, the upper interorbital distance in these bees is wider than the lower interorbital distance by about 10%, while in other *Euglossella* both distances are either equal or the lower distance is wider than the upper. Lastly, these species are restricted to the Amazon Basin and contiguous areas East of the Andes.

**Included species.** The present species group comprises *Euglossa (Euglossella) aurantia* **sp. n.**, *Euglossa (Euglossella) apiformis* Schrottky, *Euglossa (Euglossella) decorata*, *Euglossa (Euglossella) singularis* Mocsáry, *Euglossa (Euglossella) cosmodora* Hinojosa-Díaz and Engel, and *Euglossa (Euglossella) perpulchra* Moure and Schlindwein.

**Figures 1–2.**
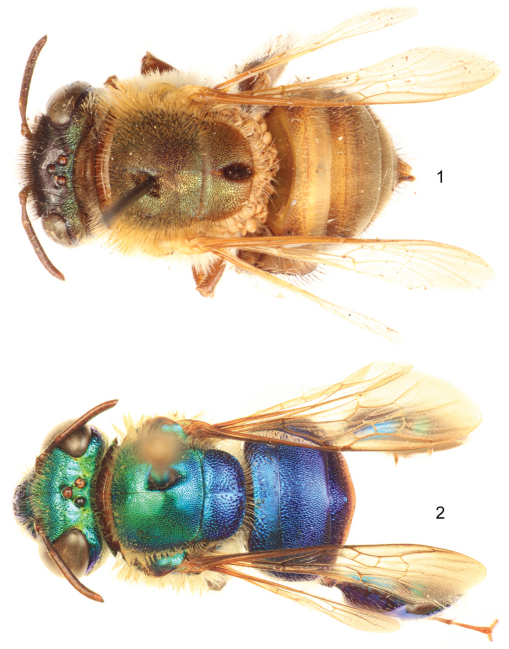
Dorsal habitus of representative species of the two species groups within *Euglossa (Euglossella)*. **1** *Euglossa (Euglossella) singularis* Mocsáry, female, *decorata* species group **2** *Euglossa (Euglossella) cyanura* Cockerell, male, *viridis* species group.

**Key to species of the *decorata* species group (males only)**

**Table:** 

1	Mesotibial tufts appearing distinct from each other, with a noticeable gap between anterior and posterior tuft; posteror tuft circular (or almost circular) (Fig. 54); clypeus with coppery/green iridescence (Guiana Shield)	*Euglossa (Euglossella) singularis* Mocsáry
–	Mesotibial tufts appearing fused at least on proximal section; posterior tuft teardrop-shaped (Figs14, 24, 44); coloration of clypeus variable .	2
2	Mesotibia with a noticeable,rather abrupt convexity on proximal area of anterior mesotibial surface, along anterior margin of anterior tuft (Fig. 13); integument of head dark brown (Bolivia)	*Euglossa (Euglossella) aurantia* sp. n.
–	Mesotibia with no noticeable convexity on proximal area along anterior margin of anterior tuft (sometimes weakly convex, but never as abrupt as in other couplet); integument of head variable	3
3	Metasoma with at least some terga exhibiting a clear banding pattern, involving either dark and light contrasting areas on individual terga, or posterior margin noticeably translucent contrasting with anterior area	4
–	Metasoma either uniformly colored or colored in a gradient, if bands present, thencolors involved are never contrasting	5
4	Second metasomal tergum with noticeably dark brown band on anterior half bordered anteriorly and posteriorly by contrasting yellow areas, remaining terga with similar pattern, sometimes hidden when metasoma is contracted (Figs 57, 59); clypeus coppery-green (Andean foothills of central Peru to Bolivia)	*Euglossa (Euglossella) cosmodora* Hinojosa-Díaz & Engel
–	Metasomal terga dark brown with posterior half noticeably translucent, forming a band pattern (Figs 66, 68); clypeus with strong coppery iridescence (northeast Brazil, Pernambuco)	*Euglossa (Euglossella) perpulchra* Moure & Schlindwein
5	Metasoma mainly dark brown with coppery iridescence; posterior margin of mesoscutellum truncate (laterally rounded) (Figs 17–20); clypeus with faint coppery iridescence (eastern Andean foothills from southern Ecuador to southern Peru)	*Euglossa (Euglossella) apiformis* Schrottky
–	Metasoma coloration generally orange-brown, some specimens dark brown; posterior margin of mesoscutellum evenly convex (Figs 35–40); clypeus with green iridescence dominant (Amazon Basin)	*Euglossa (Euglossella) decorata* Smith

**Figures 3–6.**
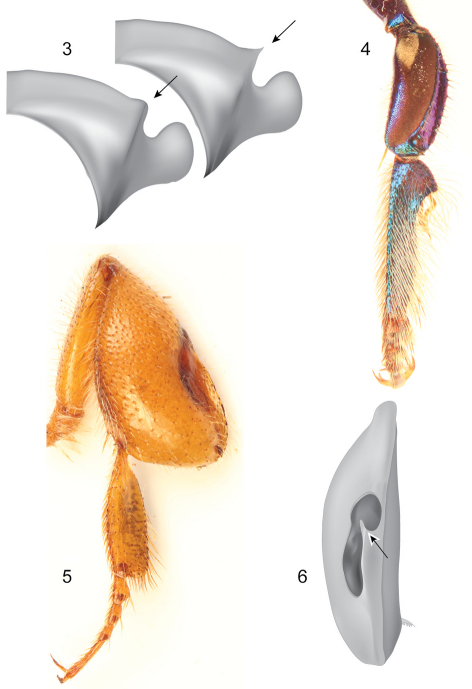
Some diagnostic features of the subgenus *Euglossella*. **3** Schematic representation of pronotal dorsolateral angle **4** Mesothoracic leg of male of *Euglossa (Euglossella) cyanura* Cockerell **5** Metathoracic leg of female of *Euglossa (Euglossella) singularis* Mocsáry **6** Schematic representation of metatibia of *Euglossa (Euglossella) decorata* Smith, showing the constriction in the metatibial organ slit.

#### Euglossa (Euglossella) aurantia sp. n.

urn:lsid:zoobank.org:act:CD07D9F6-85A9-44FF-A133-FF3F4F7C2A74

http://species-id.net/wiki/Euglossa_(Euglossella)_aurantia

Figs 7- [Fig F4] 16 

##### Holotype.

 ♂, labeled: “Bolivien, Chapare,; Rios, 11.11.2002.; leg. B. Bembé // an gelber; Solanaceae;Apocynaceae // Euglossa; decorata ♂; det. B. Bembé 2001 [second line handwritten]”. The holotype is in the Zoologische Staatssammlung München, Munich, Germany.

##### Paratype.

 ♀, labeled: “Bolivien, Chapare,; Villa Tunari, 320 m; Mai – Nov. 2002; leg. F. Heider// Euglossa; decorata ♀; det. B. Bembé 2001 [second line handwritten]”. The paratype is in the same institution as the holotype.

##### Diagnosis.

**Figures 7–8.**
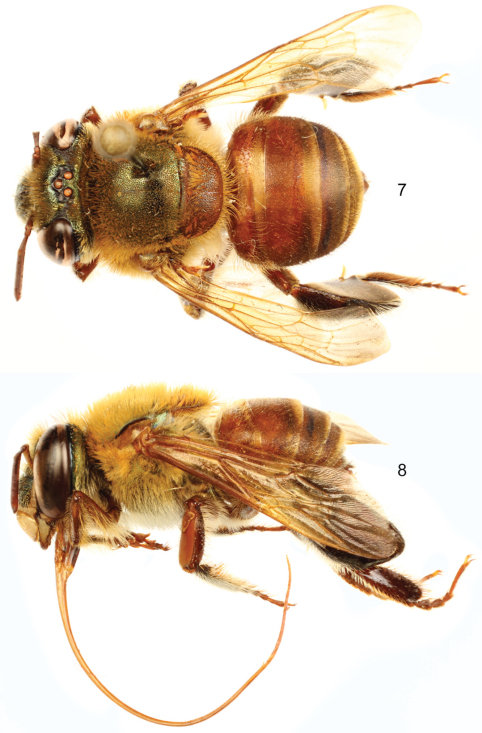
*Euglossa (Euglossella) aurantia* sp. n., male holotype. **7** Dorsal habitus **8** Lateral habitus.

**Figures 9–10. F4:**
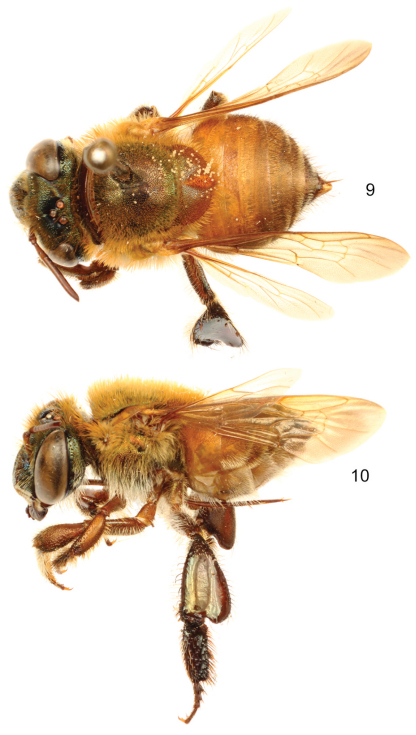
*Euglossa (Euglossella) aurantia* sp. n., female paratype. **9** Dorsal habitus **10** Lateral habitus.

**Figures 11–16.**
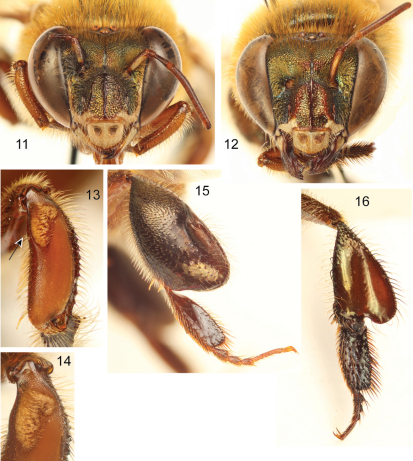
*Euglossa (Euglossella) aurantia* sp. n. **11** Facial aspect of male holotype **12** Facial aspect of female paratype **13** Outer surface of male mesotibia (arrow pointing to anterior surface convexity) **14** Mesotibial tufts **15** Outer view of male metatibia and metatarsus **16** Outer view of female metatibia and metatarsus.

 Labiomaxillary complex in repose reaching posterior tip of metasoma in the male (estimate), and posterior margin of third metasomal sternum in the female (Figs 8, 10);integument of head of both sexes dark brown to black, with green-cyan hue on frons and coppery hue on clypeus (Figs 11–12); mesosoma dark brown with green hue; mesotibia with a noticeable convexity on proximal area of anterior mesotibial surface, along anterior margin of anterior setal tuft (Fig. 13); first and second metasomal terga orange-brown, turning brown on posterolateral margins; third to seventh terga mainly brown except orange-brown on anterior margin,coppery hue iridescence on all terga; sterna orange-brown (Figs 7–10);malar area length on average 0.25 the basal mandibular width; male mesotibial tufts appearing fused (except for a distal separation),posterior tuft teardrop shaped (Fig. 14); male metatibia scalene obtuse triangular (forming a clearly obtuse angle at intersection of anterior and ventral margins) (Fig. 15).

##### Description.

♂: *Structure*. Total body length 12.44 mm; labiomaxillary complex in repose reaching posterior tip of metasoma (estimate) (Fig. 8). Head length 2.85 mm, width 5.11 mm; upper interorbital distance 2.44 mm; lower interorbital distance 2.26 mm; upper clypeal width 1.19 mm; lower clypeal width 2.19 mm; clypeal protuberance 0.81 mm; medial and paramedial clypeal ridges well developped; labrum slightly wider than long, length 1.19 mm, width 1.26 mm; medial labral ridge sharp; paramedial labral ridges noticeable but weaker than medial ridge, oblique, present in proximal three-fourths of labrum; labral windows ovoid, occupying proximal half of labrum; interocellar distance 0.30 mm; ocellocular distance 0.74 mm; first flagellomere as long (0.59 mm) as second and third flagellomeres combined (0.59 mm); length of malar area 0.19 mm. Mandible tridentate. Pronotal lateral angle projected postero-laterally as a truncate lamella (Fig. 3); intertegular distance 3.93 mm; mesoscutal length 3.04 mm; mesoscutellar length 1.48 mm; posterior margin of mesoscutellum weakly convex (Fig. 7); mesotibial length 2.59 mm, with a noticeable convexity on proximal area of anterior mesotibial surface, projected along anterior margin of anterior setal tuft; mesobasitarsal length 2.59 mm, width 0.81 mm (as measured at proximal posterior keel), posterior keel projected in a rounded orthogonal angle; metatibial shape triangular, forming a clearly obtuse angle at intersection of anterior and ventral margins (scalene obtuse triangular) (Fig. 15), metatibial anterior margin length 4.22 mm, ventral margin length 2.30 mm, postero-dorsal margin length 4.89 mm, maximum metatibial thickness 1.44 mm; metatibial organ slit dorsal and outer sections as described for subgenus; anterior margin of distal section of metatibial organ slit evenly convex, maximum width occupying slightly less than one-third of metatibial outer surface width (Fig. 15); basal section of metatibial organ slit as described for subgenus, length 0.59 mm; metabasitarsal length 2.67 mm, mid-width 0.89 mm; metabasitarsal ventral border truncate. Forewing length 10.22 mm; jugal comb with 15 blades; hind wing with 24 hamuli. Maximum metasomal width 5.19 mm; second metasomal sternum noticeably elevated mesially forming two protuberances as “false cowled slits” separated from each other by about width of labiomaxillary complex.

*Coloration*. Head mainly dark brown (except as described below), with green-cyan hue on frons and paraocular areas, mid-clypeus with coppery hue; paraocular ivory marks well developed, triangular, lower width one-half length of lower lateral parts of clypeus or slightly wider; lower lateral parts of clypeus ivory, amber-translucent at edge; labrum ivory; labral anterior and posterior edges as well as labral windows amber-translucent; malar area brown on sides (condyle, acetabulum), ivory at center; mandible ivory on basal outer surface, teeth and ridges brown; antenna light brown; scape with ivory spot covering roughly all anterior surface (Fig. 11).Pronotum, mesoscutum and propodeum dark brown with strong green hue episternum dark brown with a combination of green and coppery hue, mesoscutellum orange-brown (Figs 7–8); legs brown, turning dark brown on mesotarsomeres, metatibia and metatarsomeres, all with faint coppery hue (Figs 7–8); tegulae and wing veins light amber, hyaline, with light coppery-golden hue.First and second metasomal terga orange-brown, turning brown on posterolateral margins; third to seventh terga mainly brown, except orange-brown on anterior margin (if visible); coppery hue iridescence on all terga, appearing coppery-golden on translucent posterior sections of first to sixth terga. (Fig. 7).Sterna orange-brown, fith and sixth sterna slightly darker, posterior sections of all sterna translucent; faint coppery hue on all sterna integument.

*Sculpturing*. Face areolate-punctate, with dense, strong areole-punctures, denser and slightly smaller (nearly one-fifth of median ocellar diameter) on frons; paraocular marks and lower lateral parts of clypeus less densely sculptured; vertex moderately areolate-punctate, smooth on anterior ocellar area; gena densely areolate-punctate, smooth on a narrow streak close to compound eye (except for scattered large punctures on upper margin). Mesosoma with round, moderately-dense punctures, as big as punctures on frons; punctures separated by about one half of a puncture diameter on mesoscutum and mesepisternum, contiguous and slightly bigger on mesoscutellum (specially towards posterior margin); metatibia moderately dense punctate on antero-proximal region (along anterior margin and postero-dorsal margin previous to metatibial organ slit), becoming gradually smooth towards posterior area, especially on surface near distal section of metatibial organ slit (Fig. 15). Metasomal terga densely punctate (except smooth, polished on ventro-lateral sections and small antero-mesal surface of first tergum), puncture size comparable to that of frons punctures, increasing size ventro-laterally; metasomal sterna densely punctuate, punctures as big as ventro-lateral ones on terga, shallow, posterior margin of all sterna and contiguous areas to first sternum “false slits” smooth.

*Vestiture*. Facial setae of two kinds, some minutely branched (appearing simple), fulvous, long and sturdy, other plumose, rather fulvous, shorter and thinner. Frontal fringe with dense, fulvous, sturdy setae as long as about three mid-ocellus diameters, fulvous thin setae nearly two thirds as long as first; clypeus, supraclypeal area, and contiguous areas to clypeal disc moderately dense with an even combination of above described kinds of setae, both of about same length (about two median ocellar diameters); antennal depressions with moderately-dense, fulvous, plumose setae; paraocular marks, malar area, labrum and anterior surface of mandibles with scattered, fulvous, rather simple, short setae; vertex with scattered, fulvous, pectinate, minute setae around ocelli, interocellar area with a tuft of brown, sturdy setae; preoccipital ridge with a dense fringe comparable to the frontal one, but with brown, sturdy setae, as long as about four times median ocellar diameter; gena with dense, fulvous, plumose setae, short on upper section (where they intermix with similarly sized brown, simple, sturdy setae), increasing in length and becoming darker towards lower section, and continuing on outer mandibular margin where they become sparser, simpler and sturdier; antenna with fulvous, simple setae, long and scattered on scape, and dense and minute on flagellum. Prothorax with moderately dense fulvous, plumose, short setae; Mesoscutum, mesoscutellum and pronotal lobes covered with a combination of setae similar to that of frontal fringe, slightly longer and sturdier on pronotal lobes; mesepisternum densely covered with fulvous, plumose, long setae, becoming lighter on pleural and ventral areas; proximal podites (mainly coxae, trochanters, and part of femora) with setae as on ventral part of mesosoma; fulvous, simple, setae on femora (except as previously noted), tibiae (exceptions noted hereafter), and outer surface of tarsal articles; chemical gathering tufts on second through fourth protarsomeres made of dense, orange, long, setae; inner surfaces of probasitarsus, meso- and metatarsomeres with dense, brown, sturdy setae; mesotibia with two proximal tufts sitting on integumental concavities, anterior tuft ellipsoidal, occupying about one-third of outer tibial surface, posterior tuft teardrop shaped, slightly less than one-third as long as major axis of anterior tuft, laying on proximal posterior margin of anterior tuft, such that both tufts appear fused; both tufts made of fulvous setae directed posteriad, longer on anterior tuft (Fig. 14); microtrichia on outer mesotibial surface (velvety area) composed of dense, fulvous, simple, minute setae; anterior margin of velvety area strongly concave (Fig. 13); mesobasitarsus with three to four major wavy setae on inner surface right after proximal keel, all brown; metatibia with longer setae on anterior border and distal half of postero-dorsal margin, outer surface with scattered, brown, short, erect setae, bare on contiguous depression to metatibial organ; metatibial organ slit closed with brown setae (Fig. 15). First metasomal tergum with a mixture of setae comparable to those on posterior margin of mesoscutellum, but less dense, posterior half covered with moderately dense, fulvous, simple, minute appressed setae; second to seventh metasomal terga covered with scattered, dark brown, simple, sturdy setae as long as a median ocellar diameter, second through sixth metasomal terga with posterior bands of moderately dense, fulvous, appressed setae, as well as dense, fulvous, simple, long setal tufts on lateral margins; false slits of second metasomal sternum with tufts of moderately dense, fulvous, simple, long setae, directed posteriorly reaching posterior edge of sternum, remainder sterna with similar erect setae, mesially bare.

*Terminalia*. Genital capsule as described for subgenus. Lateral section of gonostylus with a straight dorsal sector.

♀: *Structure*. Total body length 12.22 mm; labiomaxillary complex in repose reaching posterior margin of third metasomal sternum. Head length 3.11 mm; head width 5.04 mm; upper interorbital distance 2. 59 mm; lower interorbital distance 2.37 mm; upper clypeal width 1.22 mm; lower clypeal width 2.22 mm; clypeal protuberance 0.74 mm; clypeal ridges, labral ridges and labral windows as in male; labrum rectangular, wider than long, length 1.11 mm, width 1.26 mm; anterior edge of labrum arched outwards; interocellar distance 0.37 mm; ocellocular distance 0.81 mm; length of first flagellar article (0.44 mm) equal to combined lengths of second and third flagellar articles (0.44 mm); length of malar area 0.15 mm. Mandible tridentate. Pronotal lateral angle as in male; intertegular distance 3.78 mm; mesoscutal length 3.11 mm; mesoscutellar length 1.41 mm; posterior border of mesoscutellum as in male (Fig. 9); mesotibial length 2.37 mm; mesobasitarsal length 2.30 mm, maximum width 0.74 mm; metatibia triangular; metatibial anterior margin length 3.41 mm; metatibial ventral margin length 2.07 mm; metatibial postero-dorsal margin length 3.78 mm. Forewing length 9.48 mm; hind wing with 22 hamuli. Maximum metasomal width 5.41 mm.

*Coloration*. Generally as described for male, with a mixture of coppery and green hue on face and mesosoma. Paraocular marks absent; ivory coloration on mandible restricted to proximal one-third, antennal scape with thinner yellow spot occupying upper two thirds of antero-lateral surface (Fig. 16).

*Sculpturing*. As described for male except punctures of mesepisternum less dense.

*Vestiture*. As described for male (setal features on protarsi, meso- and metatibia are exclusive of male) except as follows: Mesoscutellar tuft rhomboid, composed of dense, fulvous, erect, thick, multibranched (branches minute) setae (Fig. 19). Mesotibia with a streak of spur-like, dark brown setae on posterior and ventral edges; metatibial corbicula surrounded by long, dark brown setae. Mesial sections of all sterna nearly bare (where labiomaxillary complex resides when in repose).

##### Etymology.

 The specific epithet is a reference to the orange coloration of the metasoma in this bee species (Greek, *aurantium*, meaning “orange”).

##### Comments.

 On initial observation the two specimens here included as type material for this species look very similar to individuals of *Euglossa decorata* from the western Amazon Basin, particularly in coloration. However, aside from the generally more robust habitus of both the male and female by comparison to *Euglossa decorata*, the dominant coppery iridescence of the clypeus is notably different, which, despite a range of variation in the latter, has a consistently dominant green coloration on the clypeus. Coloration alone is not necessarily a good indication of species boundaries, so the main character that distinguishes *Euglossa aurantia* from any other species in the *decorata* group is the proximal convexity on the anterior surface of the male mesotibia along the anterior margin of the anterior mesotibial tuft (Fig. 13). Besides *Euglossa singularis*, in which this mesotibial surface is straight, all other species have a slight deviation of the integument near the distal end of the anterior margin of the anterior mesotibial tuft, but this is only appreciable at higher magnification, and does not continue as a noticeable convexity along that margin. When looking at the mesotibia of the male of *Euglossa aurantia*, the convexity in this area is immediately recognizable.

#### Euglossa (Euglossella) apiformis

Schrottky nomen revivisco

http://species-id.net/wiki/Euglossa_(Euglossella)_apiformis

**Figures 17–18.**
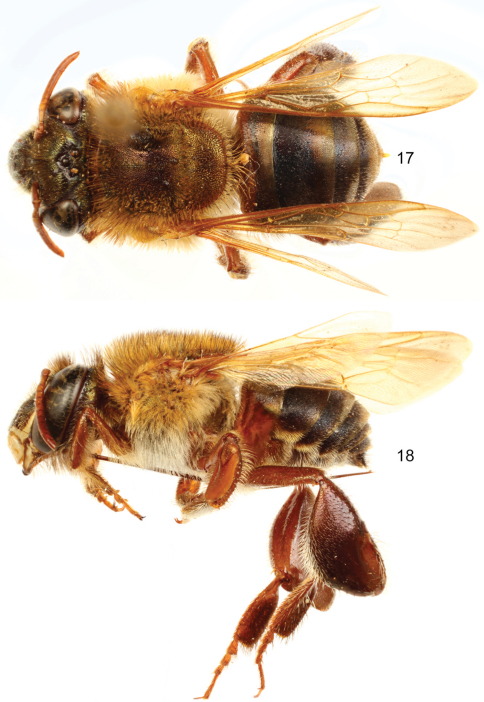
*Euglossa (Euglossella) apiformis* Schrottky, male neotype.**17** Dorsal habitus **18** Lateral habitus.

Figs 17 [Fig F7] [Fig F8] -34

Euglossa apiformis Schrottky, 1911: 39. Holotype ♀ (lost).

##### Neotype.

 ♂, labeled: “PERU: Huánuco, Llulla-; pichis [Llullapichis], Rio Pachitea; 15 II 1975 [day handwritten]; R. L. Dressler 1623 [number handwritten diagonally] // Vanillin [label upside down] // Euglossa; singularis Mocs.; det. R.L.Dressler 196”. The neotype is in the Division of Entomology, University of Kansas Natural History Museum, Lawrence, Kansas, USA.

##### Additional material.

 4♂♂, 2♀♀: labeled as follows: labeled as neotype except missing identification label (1♂) FLMNH; labeled as Neotype except date “14 II 1975 [day handwritten]” (1♂) SEMC;“PERU: Huanuco, Tingo María; Carlos Atachahua E.; 7 Aug. 1989 [day handwritten] // vanillin” FLMNH(1♂); “PERU: Madre de Dios; 30 km sw Pto. Maldonado; 1 July 1983 [day and month handwritten] M. P. Frisbie // terre firma // VANILLA [handwritten]” USNM(1♂); original collection data label as top label of Neotype except date “14 II 1975 [day handwritten]”, and diagonal handwritten number “1633” (1♀) FLMNH; “Achinamiza,; Peru I-5-26 [date handwritten]; F 6001 [number handwritten] // H.Bassler; Collection; Acc. 33591 // Euglossa; decorata Sm; Det. J.S. Moure 1957 [first two lines and last two digits of date handwritten]” (1♀) AMNH. 1♂ labeled as follows: “Ecuador: Zamora; 5-7III 1982; N. H. Williams // 89 [handwritten on the underside] // vanillin [underside]” FLMNH, this specimen is missing the head.

##### Diagnosis.

Labiomaxillary complex in repose slightly exceeding posterior tip of metasoma in the male, and posterior margin of second metasomal sternum in the female (Figs 18, 20); integument in both sexes dark brown (noticeably metasoma), with coppery-cyan hue all over (especially on clypeus), legs brown, turning dark brown on metatibia and metatarsomeres (Figs 18, 20); malar area length on average 0.25 the basal mandibular width; male mesotibial tufts appearing fused (except for a distal separation),posterior tuft teardrop shaped (Fig. 24); male metatibia scalene obtuse triangular (Fig. 25).

##### Description.

♂: *Structure*. Total body length 11.56 mm (10.74–12.74; n=5); labiomaxillary complex in repose slightly exceeding posterior tip of metasoma (Fig. 18). Head length 2.92 mm (2.73–3.11; n=5), width 4.81 mm (4.67–5.07; n=5); upper interorbital distance 2.37 mm (2.26–2.59; n=5); lower interorbital distance 2.18 mm (2.15–2.22; n=5); upper clypeal width 1.17 mm (1.11–1.19; n=5) (as measured between dorsolateral angles of clypeus); lower clypeal width 2.09 mm (2.02–2.15; n=5) (as measured at level of lower lateral parts); clypeal protuberance 0.67 mm (0.52–0.81; n=5) [following measurement method of Brooks(1988)]; clypeal ridges, labral ridges and labral windows as described for *Euglossa aurantia*; labrum slightly wider than long, length 1.13 mm (1.04–1.19; n=5), width 1.16 mm (1.11–1.20; n=5); interocellar distance 0.30 mm (n=5); ocellocular distance 0.74 mm (0.67–0.78; n=5); first flagellomere as long [0.49 mm (0.44–0.52; n=5)] as second and third flagellomeres combined [0.50 mm (0.44–0.56; n=5)]; length of malar area 0.21 mm (0.19–0.22; n=5). Mandible tridentate. Pronotal lateral angle as described for *Euglossa aurantia*; intertegular distance 3.48 mm (3.41–3.56; n=5); mesoscutal length 2.87 mm (2.81–2.96; n=5); mesoscutellar length 1.33 mm (1.26–1.41; n=5); posterior margin of mesoscutellum truncate (laterally rounded) (Fig. 17); mesotibial length 2.44 mm (2.37–2.59; n=5); mesobasitarsal length 2.56 mm (2.44–2.67; n=5), width 0.73 mm (0.67–0.79; n=5); posterior keel as described for *Euglossa aurantia*; metatibial shapeas described for *Euglossa aurantia*, metatibial anterior margin length 3.56 mm (3.41–3.85; n=5), ventral margin length 2.44 mm (2.37–2.52; n=5), postero-dorsal margin length 4.52 mm (4.37–4.59; n=5), maximum metatibial thickness 1.31 mm (1.19–1.41; n=5);metatibial organ slit dorsal and outer sections well defined with a junction noticeably narrower than contiguous width of basal section; anterior margin of distal section of metatibial organ slit evenly convex, maximum width occupying about one-third of metatibial outer surface width (Fig. 25); basal section of metatibial organ slit oval-rhomboid, length 0.64 mm (0.59–0.74; n=5); metabasitarsal length 2.57 mm (2.44–2.81; n=5), mid-width 0.85 mm (0.74–0.93; n=5); metabasitarsal ventral border truncate. Forewing length 9.78 mm (9.11–10.44; n=5); jugal comb with 13–16 (n=5) blades; hind wing with 18–23 (n=5) hamuli. Maximum metasomal width 4.77 mm (4.52–4.96; n=5); second metasomal sternum integumental modifications as described for *Euglossa aurantia*.

*Coloration*. Head similarly colored as in *Euglossa aurantia*, but with coppery-cyan hue all over (very few green highlights) (Fig. 21). Mesosoma dark brown, slightly lighter on mesoscutellar posterior margin, coppery iridescent hue throughout mesosomal integument (Figs 17–18); legs brown, slightly lighter than in *Euglossa aurantia* (Figs 18, 23–25); tegulae and wings as described for *Euglossa aurantia*. Metasomal terga dark brown, except as follows: first metasomal tergum lighter (average brown) on ventro-lateral and anterior sections, appearing even yellow in anterolateral edges; first to sixth terga with posterior margin slightly translucent; coppery iridescence on all terga, appearing coppery-golden on posterior sections of first to sixth terga. (Figs 17–18). Sterna brown, darker laterally at area of contact with terga, posterior sections of all sterna translucent; faint coppery hue on all sterna integument.

*Sculpturing*. As described for *Euglossa aurantia* (*vide supra*).

*Vestiture*.General vestiture as described for *Euglossa aurantia*, except as follows: of two kinds of setae generally present all over body, minutely branched (rather simple or serrate), sturdier ones appear darker (dark brown) than plumose ones (fulvous).

*Terminalia*. Posterior margin of seventh metasomal sternum shallowly invaginated mesally, covered with setae; eighth sternum and genital capsule as described for subgenus. Lateral section of gonostylus with dorsal sector variable, either straight or slightly projected on a hump (Figs 33–34).

**Figures 19–20. F7:**
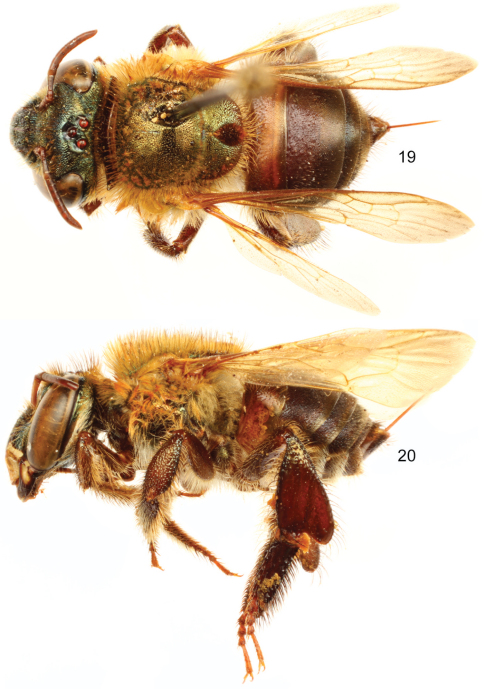
*Euglossa (Euglossella) apiformis* Schrottky, female **19** Dorsal habitus **20** Lateral habitus.

♀: *Structure*. Total body length 11.11–12.07 mm; labiomaxillary complex in repose reaching posterior margin of second metasomal sternum. Head length 2.96 mm; head width 4.74–4.81 mm; upper interorbital distance 2.48–2.52 mm; lower interorbital distance 2.25–2.30 mm; upper clypeal width 1.19 mm; lower clypeal width 2.15–2.19 mm; clypeal protuberance 0.67 mm; medial and paramedial clypeal ridges well developed; labrum rectangular, wider than long, length 1.04–1.11 mm, width 1.19–1.26 mm; labral ridges and windows as in male; anterior edge of labrum arched outwards; interocellar distance 0.33–0.37 mm; ocellocular distance 0.78–0.80 mm; length of first flagellar article (0.44–0.52 mm) equal to combined lengths of second and third flagellar articles (0.44–0.56 mm); length of malar area 0.15–0.17 mm. Mandible tridentate. Pronotal lateral angle as in male; intertegular distance 3.48–3.56 mm; mesoscutal length 2.59–2.89 mm; mesoscutellar length 1.30–1.41 mm; posterior border of mesoscutellum as in male (Fig. 19); mesotibial length 2.30–2.37 mm; mesobasitarsal length 2.15–2.37 mm, maximum width 0.70–0.74 mm; metatibia triangular; metatibial anterior margin length 3.19–3.41 mm; metatibial ventral margin length 1.85 mm; metatibial postero-dorsal margin length 3.78–3.93 mm. Forewing length 9.04–9.11 mm; hind wing with 20–22 hamuli. Maximum metasomal width 5.04–5.19 mm.

*Coloration*. In general as described for male but with a stronger coppery-cyan hue on face and metasoma. Paraocular marks absent; ivory coloration on mandible restricted to proximal one-third, antennal scape with yellow spot occupying upper half of antero-lateral surface (Fig. 22).

*Sculpturing*. As described for male except mesepisternum with punctures not as dense (separated by about one puncture diameter).

*Vestiture*. As described for male except as follows: Mesoscutum and mesoscutellar vestiture dominated by fulvous thinner setae, although dark brown kind is still present; mesoscutellar tuft rhomboid, composed of dense, fulvous and brown, erect, thick, multibranched (branches minute) setae (Fig. 19). Mesotibia with a streak of spur-like, dark brown setae on posterior and ventral edges; metatibial corbicula surrounded by long, dark brown setae. Mesial sections of all sterna nearly bare.

##### Comments.

 Schrottky (1911) described *Euglossa apiformis* from an unspecified number of females presumably from Marcapata, Cuzco, Peru (Rasmussen et al. 2010). The original description (Schrottky 1911) refers to a species in the *Euglossa decorata* species group with a dark brown metasoma and a bronze-green mesosoma, besides other characters common to all species of the group. Although some specimens of *Euglossa decorata* have a dark metasoma (see comments for *Euglossa decorata*), it usually comes with a darker mesosoma altogether, and only some *Euglossa decorata* specimens from the eastern Amazon Basin have similar coloration to the one described by Schrottky (1911) and observed in the specimens here examined. Characters not mentioned by Schrottky (1911) that distinguish this species from *Euglossa decorata* (with which it shares some distributional range) include the coloration of the clypeus being more coppery than green, a labiomaxillary complex in the male extending slightly beyond the tip of the metasoma (not surpassing it in *Euglossa decorata*), and a truncate posterior mesoscutellar margin (evenly convex in *Euglossa decorata*). *Euglossa apiformis* appears as a synonym of *Euglossa singularis* in the euglossine checklist of Moure (1967) and Kimsey and Dressler (1986), as well as in Moure et al. (2007) and Nemésio and Rasmussen (2011). This synonymy was most likely based on the assumption that any darker looking bee resembling *Euglossa decorata* would correspond to *Euglossa singularis* but as discussed later in this work this color distinction disregarded all other morphological evidence. The set of characters here presented and the locality records located in a continuous region along the lowlands contiguous to the Andes on the Amazon Basin of Peru and Ecuador justify the validity of the species. A neotype is here designated in order to validate the status of the species as described by Schrottky (1911) since the original type materialis presumed lost (Moure 1967, Kimsey and Dressler 1986, Moure et al. 2007, Rasmussen et al. 2009). The localities of the specimens here examined are in the same region and with similar elevations as the type locality (Schrottky 1911, Rasmussen et al. 2009). Although the original description was based solely on female characters, and therefore the original type corresponded to a female, a male is here designated as the neotype since males carry the most distinctive specific characters in *Euglossa* and designation of a female would carry the potential for further confusion in the future.

**Figures 21–26. F8:**
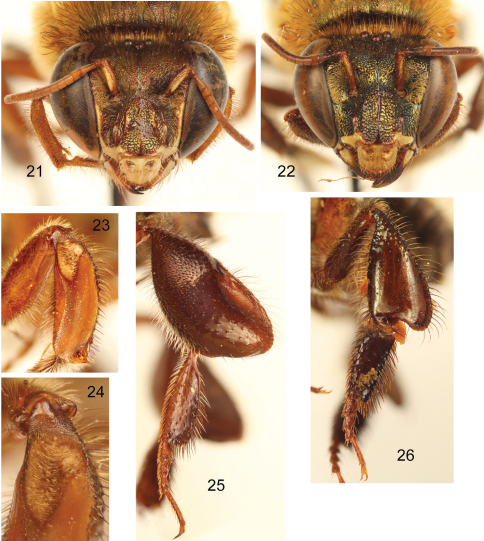
*Euglossa (Euglossella) apiformis* Schrottky **21** Facial aspect of male neotype **22** Facial aspect of female **23** Outer surface of male mesotibia **24** Mesotibial tufts **25** Outer view of male metatibia and metatarsus **26** Outer view of female metatibia and metatarsus.

**Figures 27–34.**
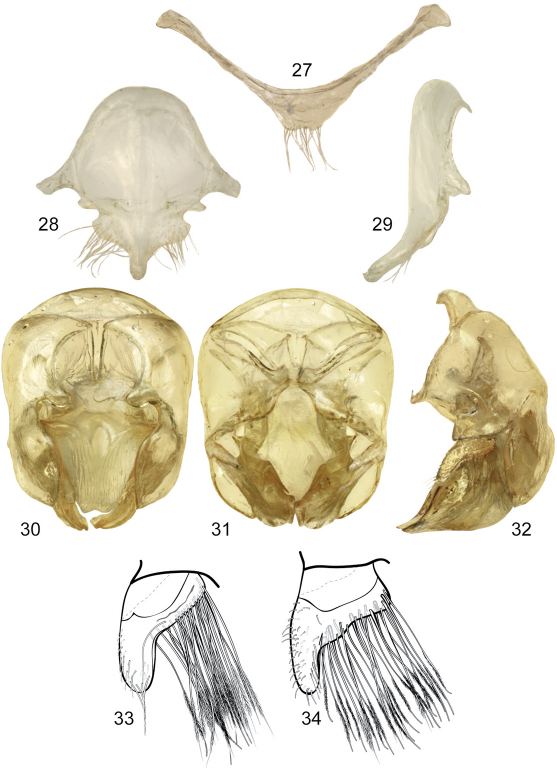
Male genitalic features of *Euglossa (Euglossella) apiformis* Schrottky **27** Seventh metasomal sternum, ventral aspect **28** Eighth metasomal sternum, ventral aspect **29** Eighth metasomal sternum, lateral aspect **30** Genitalic capsule, dorsal aspect **31** Genitalic capsule, ventral aspect **32** Genitalic capsule, lateral aspect **33** Lateral section of gonostylus, variety with straight dorsal sector **34** Lateral section of gonostylus, variety with projections on dorsal sector.

#### Euglossa (Euglossella) decorata

Smith

http://species-id.net/wiki/Euglossa_(Euglossella)_decorata

**Figures 35–36.**
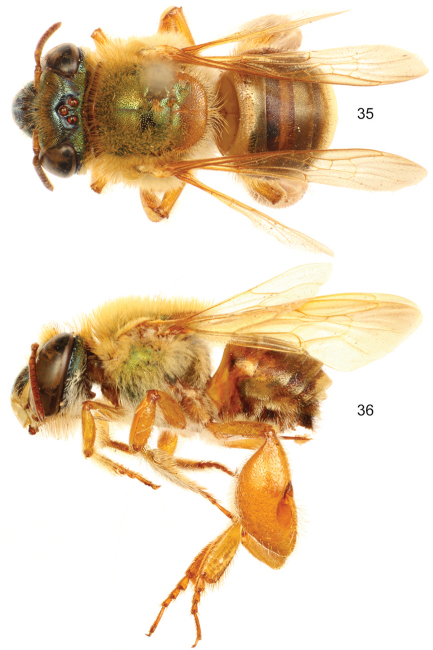
*Euglossa (Euglossella) decorata* Smith, male. **35** Dorsal habitus **36** Lateral habitus.

Figs 35- [Fig F11] [Fig F12] 46

Euglossa decorata Smith, F., 1874: 440-446 [444]. Holotype ♀ (NHML, *visum*).
Euglossa meliponoides Ducke, 1902: 569. Lectotype ♀ (MPEG, *non visum*, *vide* Comments *infra*), **syn. n.**
Euglossa (Euglossella) urarina Hinojosa-Díaz and Engel, 2007: 100-103. Holotype ♂ (FLMNH, *visum*), **syn. n.**

##### Material examined.

Colombia: “COLOMBIA: Caqueta;Yuruyaco, 73k. sw Flo-;rencia 17.i.1979 [day handwritten]; M. Cooper; B.M. 1979-106” (1♀) NHML; five extra specimens with same collection data except for date “30.i1979[day handwritten]” (1♀) BMNH, “3.ii1979[day handwritten]” (1♀) NHML, “9.ii1979[day handwritten]” (1♂) NHML “13.ii1979[day handwritten]” (1♀) NHML; “primary forest [handwritten]; COLOMBIA:Putu-;mayo, Villa Garzón,; 8mi, s. Mocoa; 17 vii.1978[day handwritten]; M. Cooper; B.M. 1978-431” (1♀) NHML; same data except missing first handwritten line and different date “19.vii.1978[day handwritten]” (1♀) NHML; “COLOMBIA:Putu-;mayo, Mocoa; 10 vii.1978[day handwritten]; M. Cooper; B.M. 1978-431” (1♂) NHML; “Colombia: Putumayo,; Mocoa, 530 m, 10 I 2003; S. Ramírez 345, V” (1♂)FLMNH; same data except number on last line “348” (1♂) FLMNH, “349” (1♂) FLMNH; “Colombia; Amazonas; Leticia; 7 VI 1974 [handwritten]; 1554 [handwritten vertical on left margin]” (1♂, missing abdomen; glued abdomen in data label does not belong to the specimen)FLMNH; same data except date “7 VI 1974 [handwritten]” (1♂) FLMNH; “Macarena Mts.; Colombia I-II-;1950. 500-650 m.; L. Bichter // Euglossa; decorata; Sm; Det. J.S. Moure 1952 [first three lines and last two digits of date handwritten]” (1♂)SEMC; same data except second label “Euglossa;decorata ♂; Sm; J.S. Moure 1963 [first three lines and last two digits of date handwritten]” (1♂) SEMC; “Villavicencia.; Columbia [Colombia]; V-28-42; W. Kamp [all label handwritten] // Euglossa; decorata; Sm; Det. J.S. Moure 1952 [first three lines and last two digits of date handwritten]” (1♂) SEMC.

Brazil: “Type; H.T. [type label, round with orange edge] // B.M. TYPE;HYM.; 17B.949 [handwritten] // Euglossa; decorata; S^t.^ Paulo: Smith. [all label handwritten] // S^t^ Paulo [underside handwritten]” (1♀) NHML; “R. Tapajós; Itaituba; 5.9.1902; Ducke [all label handwritten] // Euglossa ♂ typ.; meliponoides Ducke; det. A. Ducke. [first two lines handwritten] // Euglossa; singularis;Mocs.; Det.J.S. Moure 1957 [first three lines and last two digits of date handwritten] // Am. Mus. Nat. Hist.; Dept. Invert. Zool.; No.26003 [number handwritten] // Euglossa; meliponoides; Ducke [handwritten]” (1♂) AMNH; “Brasil Pará Con-; ceição do Araguala; 17-21 nov 1979 // Brasil Pará; W Frange” (1♀) MPEG; “Brasil; Para; 1920 [first two digit handwritten] // Euglossa; decorata; Sm.; ♂1909 Friese det. [first three lines and sex handwritten] // E. (Euglossa); decorata Sm.; J.S. Moure 57 [first two lines and two digits of date handwritten] // Am. Mus. Nat. Hist.; Dept. Invert. Zool.; No.28264 [number handwritten] (1♂) AMNH; “PA P de Pedras; 02-III-1979 [date handwritten] // Brasil Pará; P Tadeu” (1♀) MPEG; “OBIDOS; Pará BRASIL; IX-1953; F.M. Oliveira // COLECÃO; CAMPOS SEABRA [turned upside down]” (1♀) FLMNH; “TABATINGA; Amazonas BRASIL;Nov. [illegible] 1958 [date handwritten]; F.M. Oliveira // COLECÃO; CAMPOS SEABRA [underside] // decorata [handwritten]” (1♂) DZUP; “Tapuruquara – AM; Brasil VII-62; F.M. Oliveira leg” (1♂) FLMNH; “♂ // S. Gabriel; Rio Negro,; Amaz.; 27,VIII,1927; J.F. Zikán [vertical writing on left]” (1♂) FLMNH; “Belém Mocambo; 26.XII.1979 [handwritten except first two digits of year] // Brasil Pará; M F Torres” (1♀) MPEG; “Est. Ecol. do;Panga;12663 – 36987 // Uberlândia MG;BRASIL 04/02/1989;C. H. Marchini // F.90 10.12;42:89;Marchini, CH [underside, handwritten] // E. (Euglossella);decorata;Smith, 1874;Det. Camargo 1989 [first three lines and last digit of year handwritten]” (1♂) NHML.

Ecuador: “Mishaualli [handwritten]; Napo, Ecuador;D. Velastegui; 4/12/69 vanillin [handwritten] // E. singularis [handwritten]” (1♂) FLMNH; same collection data except missing identification label (1♂) FLMNH; “Choluyaco 1/7/69 [handwritten]; Napo, Ecuador;D. Velastegui; vanillin [handwritten]” (1♂) FLMNH; “ECUADOR, Coca; on Rio Napo, Napo; Pastaza Prov.; V. .1965 // L.E. Pena; Collector // Euglossa; (Euglossella); aff. singularis; det. J.S. Ascher” (1♂) AMNH; “ECUADOR: Mor.-Stgo.; E. Patuca; 27-31 Aug.;1987; Dressler, Hills,; Whitten, Williams // geraniol [underside]” (1♂) FLMNH; same data except second label “caryophylleus [unclear writing]; oxide 31” (1♂) FLMNH; “Ecuador, Orellana, Estacion; Cientifica Yasuni; to dead fish; in butterfly trap in jungle; on Sendero Napo Trail; 20Oct.; 2003;D. Robacker, W.Warfield;& M.H.Evans // Euglossa; singularis ♀; det. Claus Rasmussen 2004 [first two lines and last digit of year handwritten] // Euglossa; decorata ♀; det. Claus Rasmussen [first two lines and last digit of year handwritten]” (1♀) CRAS; “ECUADOR, Napo; September 1987; Dressler, Hills,; Whitten, Williams // vanillin” (1♂) FLMNH; same collection data without second label (1♀); “Via Tena [handwritten]; Napo, Ecuador;D. Velastegui; Nerol 1/6/1969 [handwritten] // E. decorata [handwritten]” (1♂) FLMNH; “Rio Maya 1/6/1969 [handwritten]; Napo, Ecuador;D. Velastegui; Nerol [handwritten]” (1♂) FLMNH; “Apuya 1/9/1969 [handwritten]; Napo, Ecuador;D. Velastegui; Geraniol [handwritten]” (1♂) FLMNH; “Rio Cumayacu [handwritten]; Napo, Ecuador;D. Velastegui; Nerol 3/21/1969 [handwritten] // E. decorata [handwritten]” (1♂) FLMNH; “Sarayacu 6/16/69 [handwritten]; Napo, Ecuador;D. Velastegui; Vanillin [handwritten]” (2♂♂) FLMNH; “Rio Hanzo [Anzu?] [handwritten]; Napo, Ecuador;D. Velastegui; Nerol 12/14/1968 [handwritten]” (1♂) FLMNH; “Rio Porotoyacu [handwritten]; Napo, Ecuador;D. Velastegui; MS 3-17-69 [handwritten] // Euglossa; decorata Smith” (1♂) FLMNH; “Rio Pomayaco 8/24/69 [handwritten]; Napo, Ecuador;D. Velastegui; Citronellol [handwritten]” (1♂) FLMNH; “Rio Anzo [Anzu?] 1/19/1969 [handwritten]; Napo, Ecuador;D. Velastegui; Nerol [handwritten]” (1♂) FLMNH; “Zazuyacu, Napo; Ecuador 2/12/1969;D. Velastegui; on flower [handwritten] // [second label hard to read, has some information in Spanish about the bee visiting a flower]” (1♂) FLMNH; “ECUADOR: Napo [second word handwritten]; Via Tena [handwritten]; 6 I 1969 [handwritten] // Nerol; D. Velastegui [underside, handwritten]” (1♂) FLMNH; “Satzayacu [handwritten]; Napo, Ecuador;D. Velastegui // vanillin; 9 XII 1969 [underside handwritten]” (1♂) FLMNH; “Ecuador: Pastaza; nr. Puyo 2 XI 1981; N. H. Williams // 11 [handwritten] // vanillin [underside]” (1♂) FLMNH; “Ecuador: Zamora-Ch.,; Ecuagenera, Pangüí; Williams & Whitten // at Geonoma, Whitten; 2480, QCA; 3 oct. 2003, [underside]” (1♂) FLMNH; “ECUADOR Oriente; 00°24'S, 76°36'W; Limoncocha; 25 July 1970; M. G. Naumann // Euglossa; decorata F. Smith; Det. R.L.Dressler, 1987” (1♀) SEMC.

Peru: “Iquitos; Peru // 8 Sept 64; C H Dodson // On *Gongora*; *maculata*;2734 // *Euglossa decorata* Smith // HOLOTYPE; Euglossa; urarina; I.A. Hinojosa-Díaz;& M.S. Engel [red type label]” [first three labels handwritten] (1♂) FLMNH; “Iquitos; Peru // 8 Sept 64; H Moore 20May65 // *Gongora maculata*;20May65; Helen Moore [underside]// PARATYPE; Euglossa; urarina; I.A. Hinojosa-Díaz;& M.S. Engel [yellow label]” [first three labels handwritten] (1♂) SEMC; “Iquitos; Peru // 31 Dec 64; C H Dodson // On *Gongora*;2771 // 70 // *Euglossa*; *decorata* Smith; det. R.L. Dressler 1968” [first four labels handwritten] (1♂); “Iquitos, Peru; F 606 [number handwritten // H. Bassier; Collection; Acc. 33591 // Euglossa; meliponoides; Ducke; Det. J.S. Moure 1952 [first three lines and last two digits of date handwritten] // Euglossa; singularis♀; Mocs.; J.S. Moure 1962 [first three lines and last two digits of date handwritten] // Euglossa; singularis;Mocs. (1♀) SEMC; “Lower Rio Tapiche,; Peru I.5.24 [date handwritten]; F 6/54 [numbers handwritten] // H. Bassier; Collection; Acc. 33591 // E. (Euglossa); singularis; Mocs.; J.S. Moure 57 [first three lines and digits of date handwritten]” (1♂) AMNH; “Peru, LO, Maynas,; Varillal; C.R.I. – km 15; 28 vi01Rasmussen [day handwritten] // vanillin // HYM; Euglossa; singularis; det. C. Rasmussen, 2002 [first two lines handwritten]” (1♂) CRAS; “PERU, SM, Tarapoto-; Yurimaguas, km 20; “BIODIVERSIDAD”; 0634/7620 950 masl; IV-VI.2002 C.Rasmussen // Euglossa sp.; decorata ? ♀ ; Det. Claus Rasmussen, 2002 [first two lines handwritten]” (1♀) CRAS; “PERU, Huánuco:; Tingo Mario [María], Rio; Huallaga, July 9,1974; C. Porter & L. Stange // Euglossa (Euglossella); decorata Smith, 1874; det. J.S. Ascher” (1♀) AMNH; “ Carlos Atachahua E.; 30 April 87; Tingo Maria, Peru // Vanillin // E. decorata [handwritten]” (1♂) FLMNH.

##### Diagnosis.

**Figures 37–38. F11:**
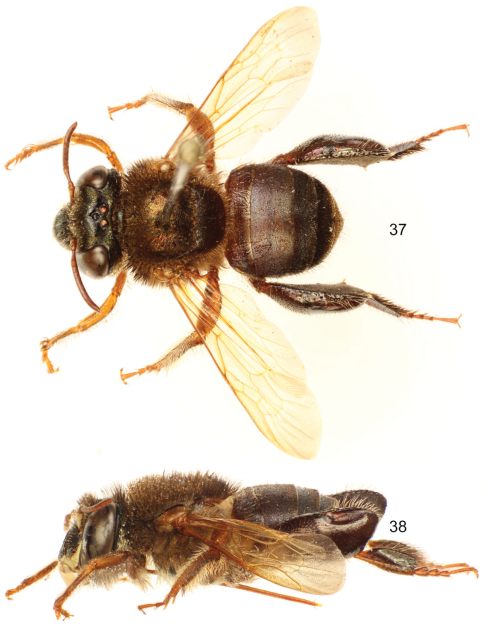
*Euglossa (Euglossella) decorata* Smith, male, dark variety, **37** Dorsal habitus **38** Lateral habitus.

**Figures 39–40. F12:**
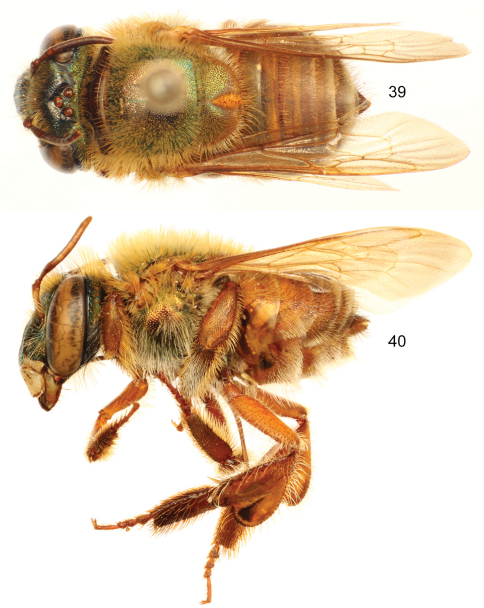
*Euglossa (Euglossella) decorata* Smith, female. **39** Dorsal habitus **40** Lateral habitus.

**Figures 41–46.**
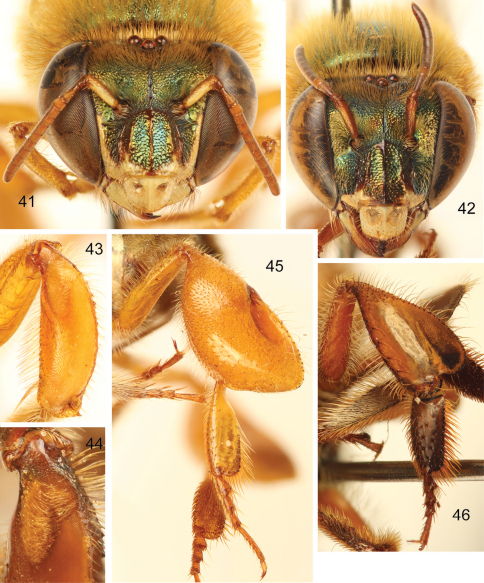
*Euglossa (Euglossella) decorata* Smith **41** Facial aspect of male **42** Facial aspect of female **43** Outer surface of male mesotibia **44** Mesotibial tufts **45** Outer view of male metatibia and metatarsus **46** Outer view of female metatibia and metatarsus.

 Both sexes with labiomaxillary complex in repose reaching tip of metasoma, but not surpassing it (Figs 36, 38, 40); head 	integument brown (variable, see comments), with a varying degree of dominant green iridescence evident on clypeus (Fig. 41); integument of mesosoma colored as head, mesoscutellum partially (see comments) light brown with diminished iridescence (Figs 35, 37, 39); metasoma generally orange-brown, terga usually darker posteriorly, coppery-golden hue all over (Figs 35-40); malar area length on average 0.20 the basal mandibular width; male mesotibial tufts appearing fused (except for a distal separation), posterior tuft teardrop shaped (Fig. 41); male metatibia scalene obtuse triangular (Fig. 45).

##### Description.

♂: *Structure*. Total body length 11.43 mm (10.30–12.59; n=7); labiomaxillary complex in repose reaching (or at most slightly exceeding) posterior tip of metasoma (Figs 36, 38). Head length 2.67 mm (2.59–2.78; n=7), width 4.63 mm (4.52–4.81; n=7); upper interorbital distance 2.28 mm (2.13–2.37; n=7); lower interorbital distance 1.99 mm (1.93–2.15; n=7); upper clypeal width 1.08 mm (1.04–1.26; n=7) ; lower clypeal width 1.94 mm (1.85–2.11; n=7); clypeal protuberance 0.71 mm (0.67–0.74; n=7); clypeal ridges, labral ridges and labral windows as described for *Euglossa aurantia*; labrum slightly wider than long, length 1.05 mm (0.98–1.11; n=7), width 1.11 mm (1.06–1.19; n=7); interocellar distance 0.27 mm (0.22–0.30; n=7); ocellocular distance 0.70 mm (0.67–0.81; n=7); first flagellomere as long [0.49 mm (0.44–0.54; n=7)] as second and third flagellomeres combined [0.48 mm (0.44–0.52; n=7)]; length of malar area 0.17 mm (0.15–0.22; n=7). Mandible tridentate. Pronotal lateral angle as described for *Euglossa aurantia*; intertegular distance 3.37 mm (3.33–3.48; n=7); mesoscutal length 2.80 mm (2.70–2.96; n=7); mesoscutellar length 1.31 mm (1.19–1.41; n=7); posterior margin of mesoscutellum evenly convex (Figs 35, 37); mesotibial length 2.37 mm (2.30–2.44; n=7); mesobasitarsal length 2.42 mm (2.37–2.52; n=7), width 0.71 mm (0.67–0.74; n=7); posterior keel as described for *Euglossa aurantia*; metatibial shape as described for *Euglossa aurantia*, metatibial anterior margin length 3.57 mm (3.26–3.70; n=7), ventral margin length 2.21 mm (2.00–2.37; n=7), postero-dorsal margin length 4.32 mm (3.85–4.67; n=7), maximum metatibial thickness 1.24 mm (1.11–1.33; n=7); metatibial organ slit as described for *Euglossa aurantia* (Fig. 45); basal section of metatibial organ slit length 0.58 mm (0.48–0.67; n=7); metabasitarsal length 2.48 mm (2.37–2.59; n=7), mid-width 0.85 mm (0.81–0.96; n=7); metabasitarsal ventral border truncate. Forewing length 9.36 mm (8.67–10.15; n=7); jugal comb with 12–14 (n=7) blades; hind wing with 20–23 (n=7) hamuli. Maximum metasomal width 4.57 mm (4.44–4.74; n=7); second metasomal sternum integumental modifications as described for *Euglossa aurantia*.

*Coloration*. Head integument brown, frons with cyan iridescence on frontal fringe area, clypeus with green iridescence, other areas with some coppery-cyan hue (see comments); ivory areas as in *Euglossa aurantia*, except lower width of paraocular marks in several specimens extending all the length of lateral parts of clypeus (Fig. 41). Mesosoma brown, mesoscutellum with at least some light brown integument usually towards posterior margin (see comments), olive-green(dominant)/coppery iridescence on mesoscutum, coppery on episternum (Figs 35–36); legs brown (variable, see comments), with a similar pattern as in *Euglossa aurantia* (Figs 36–38); tegulae and wings as described for *Euglossa aurantia* (Figs 35–38). Metasomal terga in most specimens (see comments) orange-brown, terga turning darker on poster section, sterna generally orange-brown, coppery-golden hue all over metasoma (Figs 35–38).

*Sculpturing*. As described for *Euglossa aurantia* (*vide supra*).

*Vestiture*. General vestiture as described for *Euglossa aurantia*.

*Terminalia*. Hidden sterna and capsule as described for *Euglossa apiformis*, lateral section of gonostylus variable, ranging from flat dorsal sector to large projections (Figs 33, 34).

♀: *Structure*. Total body length 11.30 mm (10.96–11.56; n=5); labiomaxillary complex in repose reaching approximately posterior margin of third metasomal sternum (Fig. 40). Head length 2.90 mm (2.78–3.04; n=5); head width 4.86 mm (4.74–4.96; n=5); upper interorbital distance 2.53 mm (2.44–2.63; n=5); lower interorbital distance 2.30 mm (2.26–2.37; n=5); upper clypeal width 1.20 mm (1.15–1.26; n=5); lower clypeal width 2.19 mm (2.15–2.26; n=5); clypeal protuberance 0.61 mm (0.52–0.67; n=5); medial and paramedial clypeal ridge well developed; labrum rectangular, wider than long, length 1.15 mm (1.07–1.19; n=5), width 1.24 mm (1.19–1.30; n=5); labral ridges and windows as in male; anterior edge of labrum arched outwards; interocellar distance 0.32 mm (0.30–0.33; n=5); ocellocular distance 0.78 mm (0.74–0.81; n=5); length of first flagellar article [0.50 mm (0.48–0.52; n=5)] equal to combined lengths of second and third flagellar articles [0.50 mm (0.47–0.52; n=5)]; length of malar area 0.17 mm (0.15–0.19; n=5). Mandible tridentate. Pronotal lateral angle as in male; intertegular distance 3.65 mm (3.56–3.78; n=5); mesoscutal length 2.95 mm (2.89–3.00; n=5); mesoscutellar length 1.40 mm (1.33–1.48; n=5); posterior border of mesoscutellum as in male (Fig. 39); mesotibial length 2.36 mm (2.30–2.44; n=5); mesobasitarsal length 2.27 mm (2.22–2.30; n=5), maximum width 0.75 mm (0.70–0.81; n=5); metatibia triangular; metatibial anterior margin length 3.27 mm (3.04–3.44; n=5); metatibial ventral margin length 1.93 mm (1.74–2.00; n=5); metatibial postero-dorsal margin length 3.64 mm (3.26–3.78; n=5). Forewing length 9.23 mm (8.74–9.70; n=5); hind wing with 21–22 hamuli. Maximum metasomal width 5.13 mm (4.96–5.26; n=5).

*Coloration*. In general as described for male but with a stronger coppery-cyan hue on face and metasoma. Paraocular marks absent; ivory coloration on mandible restricted to proximal one-third, antennal scape with yellow spot occupying most of antero-lateral surface although noticeably narrower than in male (Fig. 42).

*Sculpturing*. As described for male except mesepisternum with punctures not as dense (separated by about one puncture diameter).

*Vestiture*. As described for male (see comments); mesoscutellar tuft rhomboid, composed of dense, fulvous and/or brown (see comments), erect, thick, multibranched (branches minute) setae (Fig. 39). Mesotibia with a streak of spur-like, dark brown setae on posterior and ventral edges; metatibial corbicula surrounded by long, dark brown setae. Mesial sections of all sterna nearly bare.

##### Comments.

 Smith (1874) described *Euglossa decorata* from a female labeled as from “S^t^. Paulo”, and referred to its habitat as “St. Paulo (Brazil)”. Moure (1967) referred to the type locality as São Paulo de Olivença in the state of Amazonas, Brazil, which was repeated in Moure et al. (2007) [it is well known that old uses of S. Paulo refer to a locality in the Amazon and not today’s State or Municipality in southern Brazil: e.g., Papavero (1971)]. The majority of the specimens here examined are from the Amazon Basin, which agrees with the locality interpretation of the latter authors. Several of the distinctive features of the species are male features (as with most *Euglossa* *s. lat.*); however, the females are recognized also for the prevalence of green iridescence on the clypeus and the evenly convex mesoscutellar posterior margin. The original description of *Euglossa decorata* var. *ruficauda* by Cockerell (1918) assumed the female holotype of that variety to be conspecific with *Euglossa decorata* most likely based on coloration, which is in fact very similar in both specimens; however, as discussed later, *Euglossa decorata ruficauda* is here synonymized with *Euglossa singularis*. *Euglossa meliponoides* was synonymized with *Euglossa singularis* both by Cockerell (1918) and by Moure (1967), most likely based on the dark coloration of the specimens used in the description of these two species; however, the male of *Euglossa meliponoides* here examined and belonging to the type series exhibits the morphological features of *Euglossa decorata*, notably the mesotibial posterior tuft of the male. Interestingly, Ducke (1902),when providing the original description of *Euglossa meliponoides*, noted that his species wasvery likely just a dark variety of *Euglossa decorata*, but proposed the name in the absence of intermediate specimens in terms of coloration. The surviving holotype is a female and provides no useful characters for identification beyond color. However, we examined the paratype male from the same collecting event which clearly demonstrates the taxon to be a synonym. Given that the male exhibits more useful characters it might be worth petitioning the ICZN to have Ducke’s holotype set aside in favor of his male paratype, thereby even more strongly clarifying the status of the epithet *meliponoides*. Hinojosa-Díaz and Engel (2007) described *Euglossa urarina* as a new species in the *decorata* group addressing particularities in the male genitalia, more specifically the lateral section of the male gonostylus having a prominent dorsal projection; otherwise the specimens used by those authors had external features just like any other male of *Euglossa decorata*. As more specimens have been available for dissection of the genital capsule, it has come clear that the morphology of the lateral section of the gonostylus is highly variable in *Euglossa decorata*, ranging from simple non-projected (besides the ventral lobe), to abruptly projected as seen in the specimens described as *Euglossa urarina* (the same variation has been observed for *Euglossa apiformis*). There is no pattern of covariation with other external characters in the male that indicates at the moment a possible species-specific morphology of the gonostylus. The same can be said in terms of coloration. There is a broad range of color variation across the specimens examined for *Euglossa decorata*, most of them bearing the distinctive pattern of the holotype, with a rather golden-yellow to orange metasoma; however, all possible intermediates can be found between this and the very dark specimens from Loreto, Peru (Figs 37–38); specimens on the west range of the species seem to be darker, although not as dark as the Peruvian ones. It must be noted that wherever dark specimens occur there are also some light ones in the same habitat, and there is no major morphological difference among these. The extent of the lighter brown (turning yellowish) coloration on the mesoscutellum is also quite variable, some specimens having the whole mesoscutellum uniformly light brown or yellow (like the holotype), others having this coloration restricted to the marginal posterior edge. The vestiture color also exhibits a range of variation, correlated with the integumental coloration. The length of the labiomaxilalry complex in *Euglossa decorata* reaches the tip of the metasoma, although some females, most notably the specimen here examined from Minas Gerais, Brazil have a noticeably shorten labiomaxillary complex. Given that we could find no further distinguishing evidence, it is assumed here that these females belong to *Euglossa decorata* although we note that further review of new evidence could reveal largely cryptic species requiring recognition.

#### Euglossa (Euglossella) singularis

Mocsáry

http://species-id.net/wiki/Euglossa_(Euglossella)_singularis

**Figures 47–48.**
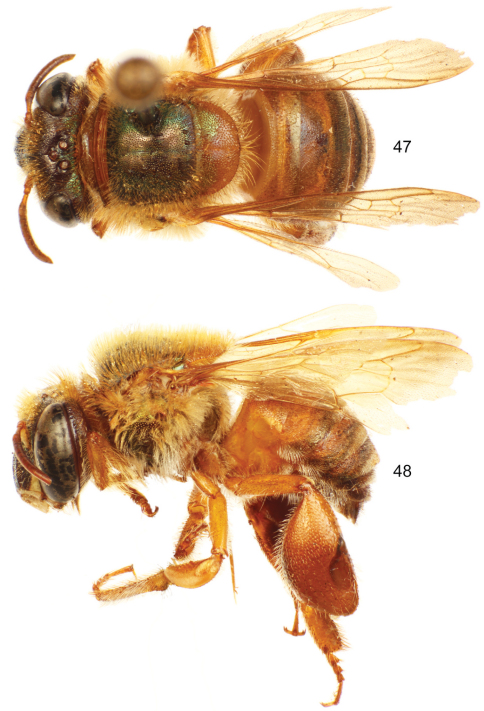
*Euglossa (Euglossella) singularis* Mocsáry, male. **47** Dorsal habitus **48** Lateral habitus.

Figs 47- [Fig F15] 56

Euglossa singularis Mocsáry *in*Friese, 1899: 169. Holotype ♀ (HNHM, *visum*).
Euglossa decorata ruficauda Cockerell, 1918: 688. Holotype ♀ (AMNH, *visum*), syn. n.

##### Material examined.

 Brazil: “SERRA do NAVIO; Terr. Amapá BRASIL; K. Lenko leg. // COLECÃO; CAMPOS SEABRA” (1♂) FLMNH.

French Guiana: “FRENCH GUIANA;Saül, Mt. Galbao Summit, 740 m; 3°37'18"N, 53°16'42"W; 6 JUN 1997; J.Ashe, R.Brooks; FG1AB97 152 // Euglossa; *decorata* F. Smith 1874 ♀ [sex handwritten]; det. R.W. Brooks 1998 // [bar code];SM0103108; KUNHM-ENT” (1♀) SEMC; “FRENCH GUIANA; 19 km. SW. Kourou;16 July 1977. C.D.; Michener, T.Kukuk” (1♀) SEMC; “FRENCH GUIANA; Kourou, Km. 17 SW. [number handwritten]; 20Feb77; D. Roubik.No.91” (1♀) FLMNH; “FRENCH GUIANA; Kourou, Km. 16 SW. [number handwritten]; 13 April 1977 [date handwritten]; D. Roubik. No.127 // *Euglossa*; *decorata* Smith; det. R.L. Dressler 1978 [last two digits handwritten]” (1♀) SEMC.

Guyana: “Kalacoon; Bartica District; British Guiana // TYPE [red label] // Am. Mus. Nat. Hist.; Dept. Invert. Zool.; No.24484 [number handwritten] // Trop. Research Station; New York Zool. Society; No.; ac: 531b [last line handwritten on underside] // Euglossa; decorata n [?]; ruficauda;Ckll. TYPE. [label handwritten]” (1♀) AMNH; “BRITISH GUIANA:; Kartabo, Bartica; Dist. 1920 [last two digits handwritten] // Trop. Research Station; New York Zool. Society; No.201122 [number handwritten] // Gift of New York; Zoo.Soc.,Dept.; Tropical Research; William Beebe. Dir // Euglossa; decorata ♀; var. ruficauda; Cockerell; Det. Schwarz // Comment on; intermixed; dark hairs on; thorax and; vertex; darker; scutellar cushion,; etc. [last two labels handwritten]” (1♀) AMNH; Kartabo; Bartica District; British Guiana; 17-III-1922 [month and day handwritten] // Gift of New York; Zoo.Soc.,Dept.; Tropical Research; William Beebe. Dir // // Euglossa; decorata ♀; var. ruficauda; Cockerell; Det. H.F. Schwarz [label handwritten] ” (1♀) AMNH; “Dawa, Tapakuma; Pomeroon, Guyana; C.Dodson 3-27-1970 [day handwritten]; Vanillin // Euglossa; decorataSmith; det. R.L. Dressler 1968 [last digit handwritten]” (1♂) FLMNH; same collecting data, no identification label (1♂) FLMNH; same collection data except date missing the day (3♂♂); “Kamakusa; Brit.Guiana; H.Lang // Euglossa; singularis; Mocs.; Det. J.S.Moure 1957 [first three lines and last two digits of year handwritten]” (1♀) NHML.

Surinam: “Amer. mer.; Surinam // Euglossa TYPE [second word handwritten]; singularis Mocs.; det. R.L.Dressler, 1975 // [big red label with no writing]” (1♀) HNHM; “[small pink label with no writing] // Surinam [handwritten] // Euglossa; singularis; ♂ Mocs.; 1910 Friese det. [first three lines handwritten, third and fourth lines overlapped] // Am. Mus. Nat. Hist.; Dept. Invert. Zool.; No.26004 [number handwritten] // Head fell off; and was reattached; by I. Hinojosa-Díaz 2006 [all handwritten except for name and first three digits of year]” (1♂) AMNH.

Venezuela: “VENEZUELA: BO. [state acronym handwritten]; Icabaru [handwritten]; 25 II 1967 [handwritten except first three digits of year] // Euglossa;singularisMocs.; det. R.L. Dressler 1968 [last digit handwritten]” (1♂) FLMNH.

##### Diagnosis.

**Figures 49–50. F15:**
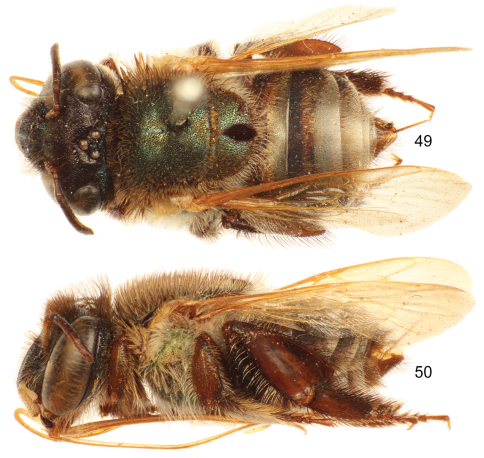
*Euglossa (Euglossella) singularis* Mocsáry, female holotype **49** Dorsal habitus **50** Lateral habitus.

**Figures 51–56.**
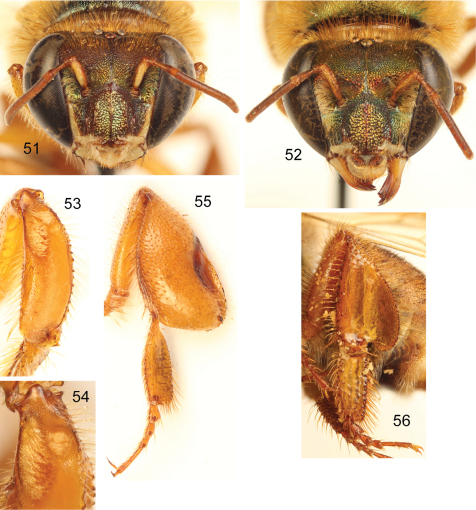
*Euglossa (Euglossella) singularis* Mocsáry **51**Facial aspect of male **52** Facial aspect of female **53** Outer surface of male mesotibia **54** Mesotibial tufts **55** Outer view of male metatibia and metatarsus **56** Outer view of female metatibia and metatarsus.

Labiomaxillary complex in repose barely reaching sixth metasomal sternum in the male, and posterior margin of third metasomal sternum in the female (Figs 48, 50); both sexes with posterior margin of mesoscutellum evenly convex (Figs 47, 49); integument of head and mesosoma of both sexes brown to dark brown, with coppery-green hue, greener on mesoscutum (Figs 47–52); malar area length on average 0.15 the basal mandibular width; male mesotibia with posterior and anterior tufts separated by a distinguishable gap, posterior tuft characteristically circular (Fig. 54); male metatibia scalene right triangular (forming a right or slightly obtuse angle at intersection of anterior and ventral margins) (Fig. 55); first metasomal tergum orange, second tergum orange anteriorly, brown on posterior third, remaining terga brown to dark brown, similar pattern on sterna (some specimens, especially females with all metasoma dark brown), entire metasoma with faint coppery hue; legs yellow to dark brown (Figs 48, 50, 53–56); lateral section of gonostylus with dorsal sector straight, not projected, ventral lobe apically acute.

##### Description.

♂: *Structure*. Total body length 10.81 mm (10.59–10.96; n=5); labiomaxillary complex in repose reaching anterior margin of sixth metasomal sternum (Fig. 48). Head length 2.73 mm (2.67–2.89; n=5), width 4.38 mm (4.22–4.48; n=5); upper interorbital distance 2.21 mm (2.19–2.22; n=5); lower interorbital distance 1.87 mm (1.81–1.93; n=5); upper clypeal width 1.01 mm (0.96–1.11; n=5) ; lower clypeal width 1.84 mm (1.78–1.89; n=5); clypeal protuberance 0.58 mm (0.44–0.67; n=5); clypeal ridges, labral ridges and labral windows as described for *Euglossa aurantia*; labrum about as wide as long, length 0.98 mm (0.96–1.04; n=5), width 1.00 mm (0.93–1.04; n=5); interocellar distance 0.3 mm (n=5); ocellocular distance 0.70 mm (0.67–0.74; n=5); first flagellomere as long [0.45 mm (0.44–0.48; n=5)] as second and third flagellomeres combined [0.45 mm (0.44–0.48; n=5)]; length of malar area 0.10 mm (0.09–0.11; n=5). Mandible tridentate. Pronotal lateral angle as described for *Euglossa aurantia*; intertegular distance 3.32 mm (3.19–3.41; n=5); mesoscutal length 2.62 mm (2.52–2.67; n=5); mesoscutellar length 1.27 mm (1.19–1.33; n=5); posterior margin of mesoscutellum evenly convex (Fig. 47); mesotibial length 2.13 mm (2.00–2.30; n=5); mesobasitarsal length 2.13 mm (2.20–2.30; n=5), width 0.62 mm (0.56–0.67; n=5); posterior keel as described for *Euglossa aurantia*; metatibial shape scalene right triangular (forming a right or slightly obtuse angle at intersection of anterior and ventral margins) (Fig. 55), metatibial anterior margin length 3.19 mm (2.93–3.41; n=5), ventral margin length 1.97 mm (1.56–2.22; n=5), postero-dorsal margin length 3.90 mm (3.78–4.15; n=5), maximum metatibial thickness 1.19 mm (1.04–1.33; n=5); metatibial organ slit as described for *Euglossa aurantia* (Fig. 55); basal section of metatibial organ slit length 0.55 mm (0.48–0.67; n=5); metabasitarsal length 2.48 mm (2.37–2.59; n=5), mid-width 0.85 mm (0.44–0.59; n=5); metabasitarsal ventral border truncate. Forewing length 8.74 mm (8.07–9.26; n=5); jugal comb with 12–15 (n=5) blades; hind wing with 18–21 (n=5) hamuli. Maximum metasomal width 4.28 mm (4.07–4.44; n=5); second metasomal sternum integumental modifications as described for *Euglossa aurantia*.

*Coloration*. Head integument and ivory areas as describred for *Euglossa decorata*, except coppery iridescence dominant on clypeus (Fig. 51). Mesosoma as described for *Euglossa decorata* (Figs 47–48); legs yellow to dark brown, with a similar pattern as in *Euglossa aurantia* (Figs 48, 53–55); tegulae and wings as described for *Euglossa aurantia* (Figs 47–48). First metasomal tergum orange, second tergum orange anteriorly, brown on posterior third, remaining terga brown to dark brown, similar pattern on sterna (some specimens, specially females with all metasoma dark brown), all metasoma with faint coppery hue (Figs 47–48).

*Sculpturing*. As described for *Euglossa aurantia*.

*Vestiture*. General vestiture as described for *Euglossa aurantia*.

*Terminalia*. Hidden sterna and capsule as described for *Euglossa apiformis*, lateral section of gonostylus with a straight or slightly convex dorsal sector (Fig. 33).

♀: *Structure*. Total body length 10.92 mm (10.00–11.63; n=5); labiomaxillary complex in repose reaching posterior margin of third metasomal sternum (Fig. 50). Head length 2.78 mm (2.67–2.85; n=5); head width 4.53 mm (4.41–4.59; n=5); upper interorbital distance 2.37 mm (2.26–2.44; n=5); lower interorbital distance 2.06 mm (2.00–2.11; n=5); upper clypeal width 1.11 mm (1.11–1.13; n=5); lower clypeal width 1.99 mm (1.93–2.00; n=5); clypeal protuberance 0.59 mm (0.52–0.67; n=5); medial and paramedial clypeal ridges well developed; labrum about as wide as long, length 1.02 mm (0.96–1.05; n=5), width 1.08 mm (1.04–1.11; n=5); labral ridges and windows as in male; anterior edge of labrum arched outwards; interocellar distance 0.30 mm (0.30–0.31; n=5); ocellocular distance 0.74 mm (n=5); length of first flagellar article [0.43 mm (0.41–0.44; n=5)] almost equal to combined lengths of second and third flagellar articles [0.45 mm (0.44–0.48; n=5)]; length of malar area 0.11 mm (0.08–0.15; n=5). Mandible tridentate. Pronotal lateral angle as in male; intertegular distance 3.33 mm (3.26–3.41; n=5); mesoscutal length 2.63 mm (2.44–2.78; n=5); mesoscutellar length 1.26 mm (1.19–1.33; n=5); posterior border of mesoscutellum as in male (Fig. 49); mesotibial length 2.19 mm (2.07–2.30; n=5); mesobasitarsal length 2.02 mm (1.93–2.15; n=5), maximum width 0.63 mm (0.52–0.70; n=5); metatibia triangular; metatibial anterior margin length 3.01 mm (2.96–3.19; n=5); metatibial ventral margin length 1.74 mm (1.56–1.93; n=5); metatibial postero-dorsal margin length 3.39 mm (3.33–3.48; n=5). Forewing length 8.34 mm (8.00–8.59; n=5); hind wing with 20–21 hamuli. Maximum metasomal width 4.67 mm (4.59–4.74; n=5).

*Coloration*. In general as described for male. Paraocular marks absent; ivory coloration on mandible restricted to proximal one-third, antennal scape with yellow spot occupying most of antero-lateral surface although noticeably narrower than in male (Fig. 52).

*Sculpturing*. As described for male except mesepisternum with punctures not as dense (separated by about one puncture diameter).

*Vestiture*. As described for male (see comments); mesoscutellar tuft rhomboid, composed of dense, fulvous and/or brown (see comments), erect, thick, multibranched (branches minute) setae (Fig. 49). Other features as described for female of *Euglossa aurantia*.

##### Comments.

 Within the variety of specimens examined in the present study, most of those that exhibited a darker coloration deviating from the orangish color of the *Euglossa decorata* type bore identification labels from several experts referring to them as *Euglossa singularis*. As was noted above for *Euglossa decorata* in which there is a range of color variation, including numerous intermediates, blending to very dark specimens, the same can be recognized for *Euglossa singularis*. Despite the fewer number of specimens of *Euglossa singularis* (as here recognized) available for this study, a similar (although not as extreme) variation of integumental coloration can be appreciated. The female holotype is the darkest of the specimens examined for this species, and the holotype of *Euglossa decorata ruficauda* is the lightest. All specimens examined, both male and female, are on average smaller than any other species in the *decorata* group and the males are easily recognizable by the shape of the mesotibial posterior tuft. The rather copperyclypeus added to the previous features, and the restriction of these specimens to the Guiana Shield region, makes *Euglossa singularis* a distinctive species, for which characterization should not rely solely on integumental color.

#### Euglossa (Euglossella) cosmodora

Hinojosa-Díaz & Engel

http://species-id.net/wiki/Euglossa_(Euglossella)_cosmodora

Figs 57–65

Euglossa (Euglossella) cosmodora Hinojosa-Díaz and Engel, 2007: 93. Holotype ♂ (SEMC, *visum*).

##### Material examined.

 Bolivia: “Bolivia; Tarata; 1900 // Euglossa; decorata; ♂ Sm.; 1909 Friese det. [first three lines handwritten, third and fourth lines overlapped] // Am. Mus. Nat. Hist.; Dept. Invert. Zool.; No.26005 [number handwritten]” (1♀) AMNH.

Peru: “PERU: Junín Dept.; Villa-Oxapampa Rd.; 1200 m 10°45'36"S, 75°21'30"W; 18 OCT 1999; R. Brooks; PERU 1B99 056; ex: on red flowering ‘Zauschneria like' // [bar code; SMO 148056; KUNHM-ENT // Euglossa; singularis ♂; Mocsáry; det. R.W. Brooks 19 [first three lines handwritten] // HOLOTYPE; Euglossa; cosmodora; I.A. Hinojosa-Díaz; & M.S. Engel [red type label]” (1♂) SEMC; same labels and data, except code on barcode label “SMO 148057”, sex on identification label “♀” and “PARATYPE” on yellow type label (1♀) SEMC; “Valle Chanchamayo; (Peru) 800 m; 5.2.1939 [day and month handwritten];leg. Weyrauch; W.K.W.; 3356 [last two lines handwritten on underside] // decorata [handwritten on underside] // PARATYPE; Euglossa; cosmodora; I.A. Hinojosa-Díaz; & M.S. Engel [yellow type label]” (1♀) DZUP; “166 [handwritten] // PERU, JU,; San Ramon; 1985; G. Arellano [label handwritten] // Euglossa; n.sp?!; det. D. Roubik 2003 [first two lines handwritten]” (1♀) CRAS.

##### Diagnosis.

**Figures 57–65.**
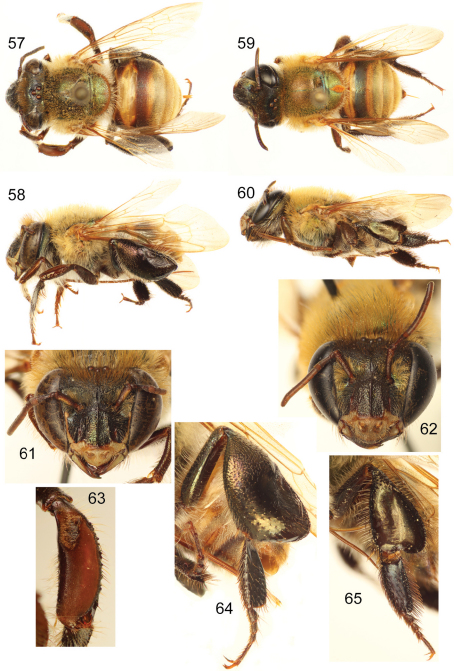
*Euglossa (Euglossella) cosmodora* Hinojosa-Díaz and Engel. **57** Dorsal habitus of male holotype **58** Lateral habitus of male holotype **59** Dorsal habitus of female paratype **60** Lateral habitus of female paratype **61**Facial aspect of male holotype **62** Facial aspect of female paratype **63** Outer surface of male mesotibia **64** Outer view of male metatibia and metatarsus **65** Outer view of female metatibia and metatarsus.

 Labiomaxillary complex in repose slightly (but clearly) surpassing metasoma (both sexes) (Figs 58, 60); both sexes with head integument very dark (appearing black), with faint coppery hue on clypeus (mixed with some green-cyan higlights) (Fig. 61); integument of mesosoma with dark brown base and strong metallic olive-green, and coppery hue (especially on episternum); metasoma golden olive-green, with a noticeably dark brown band on anterior half of second metasomal tergum bordered anteriorly and posteriorly by yellow streaks (Figs 57, 59); malar area length on average 0.30 the basal mandibular width; male mesotibial tufts appearing fused (except for a distal separation),posterior tuft teardrop shaped (Fig. 63); male metatibia scalene slightly obtuse triangular (forming a slightly obtuse angle at intersection of anterior and ventral margins) (Fig. 64).

##### Comments.

Given that a detailed description of both sexes was provided only recently by Hinojosa-Díaz and Engel (2007) and that we have no further modifications to that as presented in our earlier account, this material is not repeated here.

This species is quite distinctive, not only due to the banding pattern on the metasoma but also as it is the species withthe longest malar space of all taxa in the *decorata* species group. The specimen included herein from Bolivia extends the range of the species to the South, and is slightly lighter in coloration, although the exact locality data for this specimen is not clear (Tarata, Bolivia), the elevation of the two possible places with that locality name is clearly the highest (above 2000 m) for any specimen of this species group.

#### Euglossa (Euglossella) perpulchra

Moure & Schlindwein

http://species-id.net/wiki/Euglossa_(Euglossella)_perpulchra

Figs 66–74

Euglossa (Euglossella) perpulchra Moure and Schlindwein, 2002:586. Holotype ♂ (DZUP, *visum*).

##### Material examined.

 Brazil: “IGARASSU PE; Ref. Ecol. C. Darwin; Brasil, 21.9.2001; Schlindwein & Martini // 7753 UFPE [underside] // L121 **β**-Ionone; 9-9:30 // HOLOTYPUS ♂; Euglossa; perpulchra; Pe J. S. Moure 2001 [red label; sex, second and third line, and year handwritten]” (1♂) DZUP; “IGARASSU PE; R. E. Charles Darwin; Brasil, 20.03.2001; P. Martini leg. // L121; (1) **β**Ionone; 08:00-08:30 // 5415 UFPE // Euglossa (Euglossella); perpulchra Moure &; Schlindwein 2002 ♂” (1♂) SEMC; same data and labels except date “19.11.2000” and number on third label “3914 UFPE” (1♂) NHML; “IGARASSU PE; Ref. Ecol. C. Darwin; Brasil, 21.9.2001; Schlindwein & Martini // L121 **β**-Ionone; 10-10:30 // 7161 UFPE // PARATYPUS; Euglossa ♂; perpulchra; Pe J. S. Moure 2001 [second and third line, and year handwritten]” (1♂) DZUP; “CAMARAGIBE PE; Aldeia; Brasil, 29.5.2002; C. Schlindwein leg. // 8319 UFPE // L120 P541; 7:50; Tecoma stans” (1♀) DZUP; same data except time “7:30” (1♀) DZUP.

##### Diagnosis.

**Figures 66–74.**
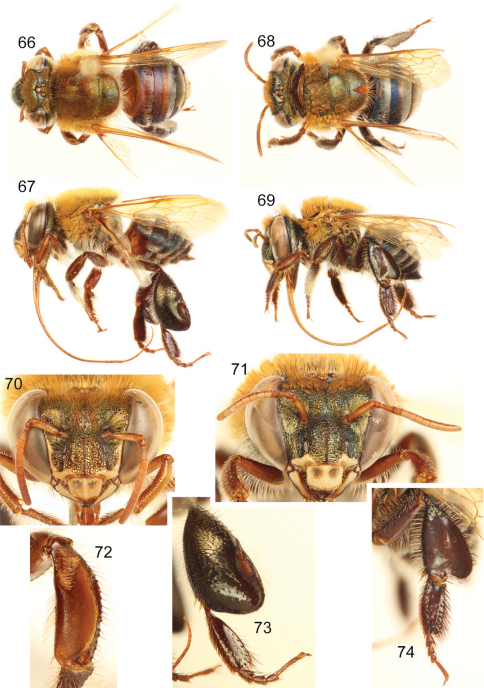
*Euglossa (Euglossella) perpulchra* Moure and Schlindwein. **66** Dorsal habitus of male paratype **67** Lateral habitus of male paratype **68** Dorsal habitus of female **69** Lateral habitus of female.**70** Facial aspect of male paratype **71** Facial aspect of female. **72** Outer surface of male mesotibia **73** Outer view of male metatibia and metatarsus **74** Outer view of female metatibia and metatarsus.

 Both sexes with labiomaxillary complex in repose nearly reaching metasomal posterior tip (estimation) (Figs 67, 69); integument of head very dark (appearing black) with strong coppery iridescence on clypeus, and green iridescence on frons (Figs 70–71); mesosoma dark brown (appearing black in most parts) with strong coppery iridescence intermixed with some cyan iridescence (Figs 66–69); metasoma dark brown (appearing black in some parts), all terga (except last) with posterior half noticeably translucent, forming a band pattern, all metasoma with cyan-coppery hue (Figs 66–69); malar area length on average 0.25 the basal mandibular width; male mesotibial tufts appearing fused (except for a distal separation), posterior tuft teardrop shaped (Fig. 72); male metatibia scalene slightly obtuse triangular (Fig. 73).

##### Comments.

Given that a detailed description for the species has been published relatively recently (Moure and Schlindwein 2002), we have not repeated that material herein. The only additions needed are that the male terminalia, unfortunately not examined or discussed by Moure and Schlindwein (2002),are as described for *Euglossa apiformis* in terms of the hidden sterna, while the genital capsule, and particularly the gonostylus, is as described for *Euglossa aurantia*. The female was also not known at the time of the original description (Moure and Schlindwein 2002). We were able to examine two female specimens in the course of this study. The female exhibits basically the same features as the male (*i.e*., coloration, punctation, and vestiture), besides having antennae light-brown with a small yellowish spot on the upper anterior surface of the scape, and the regular features observed in other females of the species group (Figs 68–69, 71, 74).

## Discussion

Prior to the the description of *Euglossa perpulchra* and *Euglossa cosmodora* (Moure and Schlindwein 2002; Hinojosa-Díaz and Engel 2007), this group of bees had been regarded as consisting of merely two species, vaguely separated by integumental coloration. Specimens with a generalized light color were assigned to *Euglossa decorata*, while any specimen showing some darkening of the integument, mostly on the metasoma, was assigned to *Euglossa singularis*. Two other dark colored forms – *Euglossa apiformis* and *Euglossa meliponoides* (Schrottky 1911; Ducke 1902) – were considered synonyms of *Euglossa singularis*.Herein we reinterpret the group to include at least six distinctive species based on a combination of external characters that is concordant with distributional ranges. The scarcity of specimens for the group makes it likely that as more of them become available and additional characters are added, more species will be recognized. It is interesting to note the ranges of color and gonostylar variation within individual populations of some species, variations not correlated with each other nor with any other structural features. The maintenance of such variation might serve some function but it is entirely obscure at present. Population genetic studies on *Euglossa decorata* and *Euglossa singularis* would be fascinating, although the relative rarity of these bees is at present a hindrance to such work. It is possible that *Euglossa decorata* is a broad-ranging species with considerable variation (as we have herein conceived) and that it has given rise to peripheral isolates which eventually formed the other species in the group [e.g., perhaps via modes similar to ones suggested by Mayr (1954, 1959) or Brown (1957)]. For the time being, we hope that this brief contribution will highlight what we believe to be congruent patterns among evolutionary species of the *decorata* group, and spur continued collection and field investigation into this highly unique lineage of *Euglossella*, and *Euglossa* as a whole.

**Figure 75.**
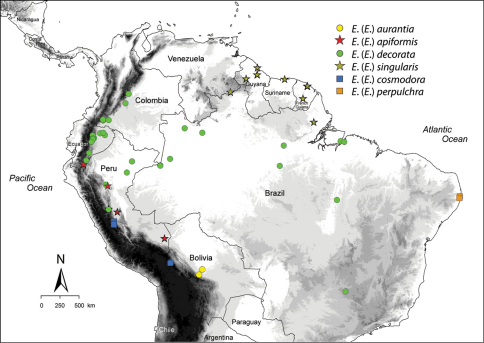
Collection localities for material examined of the six species of *Euglossella* considered herein.

## Supplementary Material

XML Treatment for Euglossella

XML Treatment for Euglossa (Euglossella) aurantia

XML Treatment for Euglossa (Euglossella) apiformis

XML Treatment for Euglossa (Euglossella) decorata

XML Treatment for Euglossa (Euglossella) singularis

XML Treatment for Euglossa (Euglossella) cosmodora

XML Treatment for Euglossa (Euglossella) perpulchra
